# Ultra-Reliable and Low-Latency Wireless Hierarchical Federated Learning: Performance Analysis †

**DOI:** 10.3390/e26100827

**Published:** 2024-09-29

**Authors:** Haonan Zhang, Peng Xu, Bin Dai

**Affiliations:** 1 School of Information Science and Technology, Southwest JiaoTong University, Chengdu 611756, China; zhanghaonan@my.swjtu.edu.cn; 2 Chongqing Key Laboratory of Mobile Communications Technology, Chongqing 400065, China; xupeng@cqupt.edu.cn; 3 Provincial Key Lab of Information Coding and Transmission, Southwest Jiaotong University, Chengdu 611756, China; 4 School of Communications and Information Engineering, Chongqing University of Posts and Telecommunications, Chongqing 400065, China

**Keywords:** finite block-length coding, physical layer security, privacy-utility relationship, wireless federated learning

## Abstract

Wireless hierarchical federated learning (WHFL) is an implementation of wireless federated Learning (WFL) on a cloud–edge–client hierarchical architecture that accelerates model training and achieves more favorable trade-offs between communication and computation. However, due to the broadcast nature of wireless communication, the WHFL is susceptible to eavesdropping during the training process. Apart from this, recently ultra-reliable and low-latency communication (URLLC) has received much attention since it serves as a critical communication service in current 5G and upcoming 6G, and this motivates us to study the URLLC-WHFL in the presence of physical layer security (PLS) issue. In this paper, we propose a secure finite block-length (FBL) approach for the multi-antenna URLLC-WHFL, and characterize the relationship between privacy, utility, and PLS of the proposed scheme. Simulation results show that when the eavesdropper’s CSI is perfectly known by the edge server, our proposed FBL approach not only almost achieves perfect secrecy but also does not affect learning performance, and further shows the robustness of our schemes against imperfect CSI of the eavesdropper’s channel. This paper provides a new method for the URLLC-WHFL in the presence of PLS.

## 1. Introduction

Wireless federated learning (WFL), which allows the training of machine learning (ML) models on a large corpus of decentralized data stored on mobile devices [1], has attracted significant research interest. Currently, WFL primarily aims to enhance communication efficiency [2,3,4,5,6], improve privacy and security [7,8,9], investigate a balance between privacy and utility [10,11,12,13,14], manage power control for wireless devices [15,16], and design effective beamforming strategies [17]. To optimize the processing power of edge and cloud servers, a hierarchical federated learning (HFL) system involving clients, edge servers, and cloud servers has been proposed in [18]. Compared to FL systems relying on a single server, HFL reduces the computational load [19,20,21], lowers user-to-cloud server communication costs [18,19,20,21,22,23], decreases FL processing time [18], and improves privacy and security in FL [22,23]. Specifically, since the convergence performance of the HFL system is theoretically proved in [18], joint user scheduling and wireless resource allocation are established to improve both communication and energy efficiency [19,20,21]. To enhance privacy in wireless hierarchical federated learning (WHFL), ref. [22] introduced a method based on local differential privacy (LDP) that involves artificial noise into the shared model parameters at two stages. Additionally, ref. [24] considered the influence of device mobility on the learning performance of WHFL systems.

The broadcast nature of wireless communication renders WFL susceptible to eavesdropping. As a result, tackling the challenge of WFL in the presence of physical layer security (PLS) is a significant issue. Different from the privacy requirement of the FL that the information leakage between users and servers does not need to be arbitrarily small due to the accuracy of data analysis, the information leakage to the eavesdropper should vanish, which is also known as the PLS requirement [25]. The current research in WFL in the presence of PLS [26,27,28,29] primarily focuses on enhancing the security of data through resource allocation and artificial jamming techniques. Specifically, reference [26] focuses on optimizing the power control of drones to enhance the security rate of the WFL system, considering constraints such as WFL training time and battery capacity of the drone. In [27], a method for achieving secrecy in WFL was proposed via using cooperative jamming, which involves the cooperative provision of jamming signals by users to counteract eavesdropping attempts and enhance security. Ref. [28] proposed the method of using conventional wireless devices to form a non-orthogonal multiple access (NOMA) transmission group with an edge device for secrecy-enhanced mobile edge computing, and the devices provide cooperative jamming to an eavesdropper while transmitting data to a cellular base station. In [29], a power allocation algorithm is proposed for WFL, where the transmitting power is divided proportionally between the transmitted signal and artificial noise to maximize the secrecy rate while satisfying the model performance requirement. Apart from this, ref. [30] proposed a PLS measure while considering the privacy-utility constraints in WFL.

Very recently, ultra-reliable and low-latency communication (URLLC) has attracted significant attention, as it serves as a critical communication service in fifth-generation (5G) and sixth-generation (6G) cellular networks. One essential technology for URLLC is short-sized packet communication [31], which indicates that the coding block length should be finite, and finite block-length (FBL) coding [32] provides an effective way for this scenario. Currently, the study of WFL combined with URLLC includes the design of a multi-level architecture to satisfy URLLC requirements [33], and the application of WFL in vehicular networks while considering URLLC constraints [34]. To the best of the authors’ knowledge, the practical FBL scheme for the WFL remains unknown. Then it is natural to ask: *is there any practical FBL scheme for the WHFL in the presence of PLS, if yes, what is the relationship between PLS, privacy, and utility in WHFL systems while considering URLLC requirements?*

One possible solution to the aforementioned question is a channel feedback coding scheme. The study of channel feedback scheme started from [35], where an elegant feedback coding scheme called the Schalkwijk–Kailath (SK) scheme was proposed for additive white Gaussian noise (AWGN) channel with noiseless feedback. In this scheme, the transmitter sends the original message only in the initial transmission. In the subsequent round, the receiver sends an estimate of the original message to the transmitter via a noiseless feedback channel. The transmitter sends an amplified version of the estimation error back to the receiver, and the receiver obtains an estimate of the estimation error by using the minimum mean square error (MMSE). After a predetermined number of rounds, the receiver uses the minimum distance rule to decode the message. It was shown that the SK scheme [35] is not only capacity-achieving but also its decoding error probability *doubly exponentially decays* to zero as the coding block length increases, which indicates that the SK scheme requires an extremely short coding block length to achieve a desired decoding error probability. Furthermore, ref. [36] showed that the SK scheme achieves perfect weak secrecy by itself, i.e., the SK scheme satisfies PLS requirement by itself. Recently, ref. [37] showed that the SK scheme [35] is almost the optimal FBL scheme for the AWGN channel with feedback, which indicates that it may be a good choice for URLLC.

However, note that the application of the SK scheme to the wireless fading channel still has a long way to go since it is based on the assumption that the feedback channel is a noiseless channel. Apart from this, in wireless communication, the channel feedback is often utilized to transmit channel state information (CSI) back to the device for each uplink transmission [17], and this allows the device to adjust its transmission parameters based on the received feedback. Then it is natural to ask: Can we utilize the channel feedback not only for CSI transmission but also for designing an FBL approach for the multi-antenna URLLC-WHFL in the presence of PLS, i.e., is it possible to extend the classical SK scheme to the multi-antenna URLLC-WHFL in the presence of PLS?

**Figure 1:**
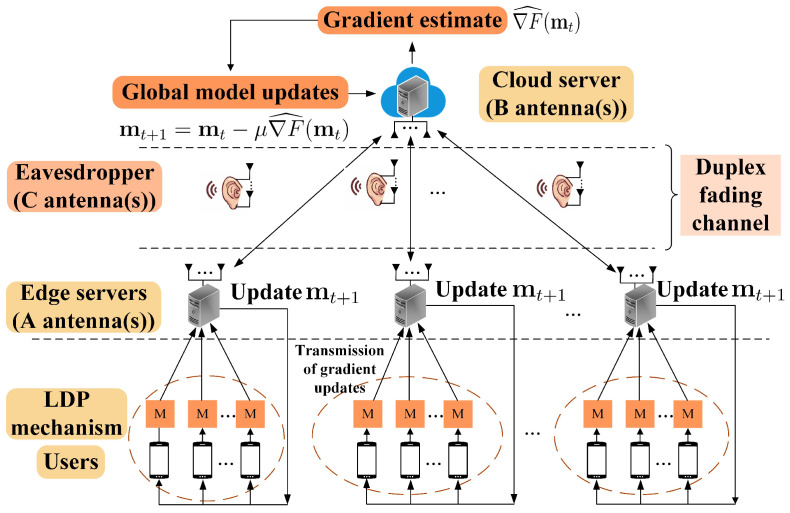
The multi-antenna WHFL in the presence of PLS.

In this paper, we answer the aforementioned questions by studying the WHFL in the presence of PLS. Figure 1 illustrates the collaborative training of a learning model by users, edge servers, and cloud servers. To preserve privacy, a local differential privacy (LDP) mechanism [38] is utilized by adding Gaussian noise to each user’s gradient before aggregating all gradients to the edge servers. Furthermore, communication between each edge server and the cloud server over a quasi-static fading duplex channel, which, due to the inherent broadcast characteristics of wireless communication, is eavesdropping by an external eavesdropper. Our primary objective is to ensure that the polluted gradient data retains a certain amount of utility while minimizing privacy leakage to the cloud server and protecting the gradient data transmitted from edge servers to the cloud server from eavesdropping. A straightforward way to achieve the above goal is for the edge servers to securely encode the polluted data gradients as codewords and transmit them into wireless duplex fading channels. The cloud server can successfully decode the polluted data gradients, while the eavesdropper obtains no information about them. In this way, the PLS and the privacy of the data can simultaneously be guaranteed since the real data gradients are protected by the LDP mechanism.

Our key contributions to this paper are summarized as follows:

- We propose an FBL approach for multi-antenna WHFL in the presence of PLS. In this approach, the feedback link is not only utilized for CSI transmission but also used to send the cloud server’s MMSE about the transmitted polluted data gradient back to the edge server. The key idea of the proposed scheme is to apply the modulo-lattice operation (MLO) [39] to eliminate the impact of feedback channel noise on the performance of the SK scheme [35], and further extend the SK-type scheme to a two-dimensional situation, which performs well in the SISO fading channel. Then further applying pre-coding, beamforming, and singular value decomposition (SVD) techniques to the extended scheme for the SISO case, the FBL coding scheme for the multi-antenna WHFL is obtained.
- We derive the achievable secrecy rate of our proposed scheme and characterize the relationship between PLS, privacy, and utility of our scheme. Moreover, given fixed decoding error probability and coding block length, we establish lower and upper bounds on the LDP noise variance that ensure certain privacy, utility, and secrecy levels of PLS.

To obtain a better understanding of the contribution of this paper and the related works studied in the literature, the following Table 1 summarizes the study of WFL in the presence of privacy, utility, PLS, and URLLC in the literature.

The remainder of this paper is organized as follows. In Section 2, the definitions, system model, and main results are given. The FBL approach for the MIMO case is shown in Section 3. FBL approaches for the SIMO/MISO cases are proposed in Section 4. Simulation results are shown in Section 5. Section 6 summarizes all results in this paper and discusses future work.

## 2. Definitions, System Model and Main Results

### 2.1. WHFL System

Figure 1 illustrates a system composed of $K_{\mathrm{tot}}$ users, *L* edge servers indexed by *ℓ* and a cloud server. The disjoint user sets are denoted as ${\left\{C_{\ell }\right\}}_{\ell =1}^{L}$, and $K=\;|C_{\ell }|$ representing the number of users in edge server *ℓ*. The distributed datasets are represented by ${\left\{S_{\ell ,k}\right\}}_{k=1}^{|C_{\ell }|}$, where $S_{\ell ,k}=\left|S_{\ell ,k}\right|$ is the size of $S_{\ell ,k}$. Each dataset $S_{\ell ,k}$ is defined as ${\left\{\left(u_{k,j},v_{k,j}\right)\right\}}_{j=1}^{|S_{\ell ,k}|}$, where $u_{k,j}$ represents the *j*-th input sample and $v_{k,j}$ is the corresponding label. $S_{\ell }$ is the aggregated dataset of edge server *ℓ*, and the gradients from each user are aggregated by their corresponding edge server. The global loss function F(m) is defined as follows:

(1) $$\begin{array}{c} F\left(m\right)=\frac{1}{S}\sum_{\ell =1}^{L}\sum_{k=1}^{|C_{\ell }|}S_{\ell ,k}F_{\ell ,k}\left(m\right), \end{array}$$

where model vector $m\in R^{q}$ and $S=\sum_{\ell }\sum_{k}S_{\ell ,k}$. The local loss function is given by the following:

(2) $$\begin{array}{c} F_{\ell ,k}\left(m\right)=\frac{1}{S_{\ell ,k}}\sum_{\left(u_{k,j},v_{k,j}\right)\in S_{\ell ,k}}f\left(m;u_{k,j},v_{k,j}\right), \end{array}$$

where $f(m;u_{k,j},v_{k,j})$ represents the sample-wise loss function. The goal of model training is to minimize the global loss function, as follows:

(3) $$\begin{array}{c} m^{★}=\arg\min_{m}F\left(m\right). \end{array}$$

To achieve this, we employ a distributed gradient descent iterative algorithm. Specifically, in the *t*-th (t∈{1,2,…,T}) communication round, the cloud server broadcasts the current global model vector $m_{t}$ to all users, and every user has perfect knowledge of $m_{t}$. Each user *k* then computes its local gradient $\nabla F_{\ell ,k}\left(m_{t}\right)$ using its dataset $S_{\ell ,k}$ and the current model $m_{t}$. Once the edge server *ℓ* receives all the noisy local gradients from its users, which have been perturbed by Gaussian noise for LDP, it computes an estimation of the partial gradient as follows:

(4) $$\begin{array}{c} \nabla F_{\ell }\left(m_{t}\right)=\frac{1}{S_{\ell }}\sum_{k\in C^{\ell }}S_{\ell ,k}\nabla F_{\ell ,k}\left(m_{t}\right), \end{array}$$

where $S_{\ell }=\left|S_{\ell }\right|$ denotes the size of $S_{\ell }$. Then, the cloud server aggregates the partial gradient estimates from all edge servers to compute the estimation $\hat{\nabla F}\left(m_{t}\right)$ of the global gradient, as follows:

(5) $$\begin{array}{c} \nabla F\left(m_{t}\right)=\frac{1}{S}\sum_{\ell =1}^{L}S_{\ell }\nabla F_{\ell }\left(m_{t}\right), \end{array}$$

and updates the global model $m_{t+1}$ by the following:

(6) $$\begin{array}{c} m_{t+1}=m_{t}-\mu \hat{\nabla F}\left(m_{t}\right), \end{array}$$

where μ denotes the learning rate.

### 2.2. Model Formulation

**Figure 2:**
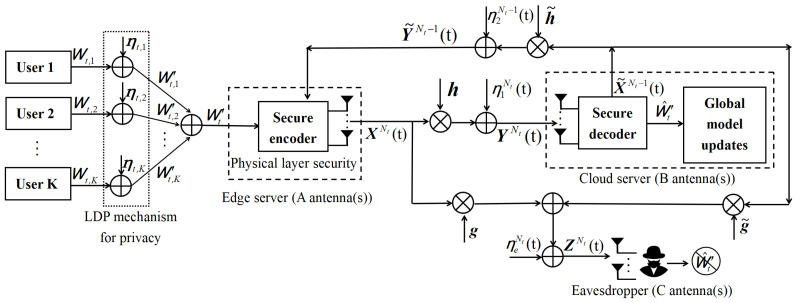
An information-theoretic model of the WHFL system, where the edge server, cloud server and eavesdroppers are equipped with *A*, *B*, and *C* antennas, respectively (A≥1,B≥1,C≥1).

An information-theoretic model of WHFL system is shown in Figure 2. Without loss of generality, we adopt the following assumptions:

**Assumption** **1.** *The communication of any individual edge server to the cloud server is not affected by other edge servers, and the downlink transmission from the cloud server to the edge servers is reliable [16]. Furthermore, we consider that an external eavesdropper targets the information transmitted during the uplink communication from the edge servers to the cloud server. Consequently, this paper primarily focuses on the PLS of the T rounds of uplink communication from one edge server to the cloud server.*

**Assumption** **2.** *The channels are quasi-static fading.*

**Assumption** **3.** *Following similar arguments in [3,9,16,17], we assume that the perfect CSI of the feedforward and feedback channels is known by both the cloud server and the edge server. Here note that this assumption is well-justified from a practical standpoint. For the feedforward channel, the channel training for estimating CSI at the cloud server can be achieved by transmitting pilot sequences from the edge servers, and the channel estimation is perfect when the length of the pilot sequences is sufficiently large [40]. On the other hand, when the cloud server transmits the perfectly estimated CSI to the edge server through the feedback channel, only a few feedback bits are required. By using a code with a low coding rate and high error-correcting capability, the probability of feedback error can be negligible [41] and, hence, the CSI of the feedforward channel is perfectly known by the transceiver. For the feedback channel, the perfect CSI sharing between transceivers can be realized in a similar way.*

#### 2.2.1. Privacy-Utility

In Figure 2, let $W_{t,k}=\sum_{j=1}^{S_{\ell ,k}}\nabla f\left(m_{t};u_{k,j},v_{k,j}\right)={\left(W_{t,k,1},\ldots ,W_{t,k,q}\right)}^{T}\in R^{q}$ represent the overall local gradient vector for user *k* (k∈{1,2,…,K}) during the *t*-th (t∈{1,2,…,T}) communication round, where $\nabla f\left(m_{t};u_{k,j},v_{k,j}\right)={\left(\nabla f_{1}\left(m_{t};u_{k,j},v_{k,j}\right),\ldots ,\nabla f_{q}\left(m_{t};u_{k,j},v_{k,j}\right)\right)}^{T}$ and $W_{t,k,i}=\sum_{j=1}^{S_{\ell ,k}}\nabla f_{i}\left(m_{t};u_{k,j},v_{k,j}\right)$ (i∈{1,2,…,q}). Following [42], assume that $\nabla f(m_{t};$$u_{k,j},v_{k,j})$ is independent and identically distributed (i.i.d.) and $\nabla f(m_{t};$$u_{k,j},v_{k,j})∼N\left(0,\sigma_{w,t}^{2}I\right)$, which indicates that $W_{t,k}∼N\left(0,S_{\ell ,k}\sigma_{w,t}^{2}I\right)$. The i.i.d. generated local Gaussian noise $\eta_{t,k}={\left(\eta_{t,k,1},\ldots ,\eta_{t,k,q}\right)}^{T}$ follows distribution $N(0,\sigma^{2}I)$ and is independent of $W_{t,k}$. The edge server aggregates the corrupted local gradient, and it is defined as follows:

(7) $$\begin{array}{ccc} & & W_{t,k}^{\prime }=W_{t,k}+\eta_{t,k}, \end{array}$$

where $W_{t,k}^{\prime }∼N\left(0,\left(S_{\ell ,k}\sigma_{w,t}^{2}+\sigma^{2}\right)I\right)$. The overall local gradients and noise for the *t*-th round are $W_{t}={\left(W_{t,1},\ldots ,W_{t,q}\right)}^{T}$ and $\eta_{t}={\left(\eta_{t,1},\ldots ,\eta_{t,q}\right)}^{T}$, respectively, where $W_{t,i}=\sum_{k=1}^{K}W_{t,k,i}$, $\eta_{t,i}=\sum_{k=1}^{K}\eta_{t,k,i}$ and i∈{1,2,…,q}. Consequently, from (7), the overall corrupted local gradients for the *t*-th round are $W_{t}^{\prime }={\left(W_{t,1}^{\prime },\ldots ,W_{t,q}^{\prime }\right)}^{T}$, where $W_{t,i}^{\prime }=\sum_{k=1}^{K}W_{t,k,i}^{\prime }$ and i∈{1,2,…,q}. Due to the fact that $W_{t,k}$ and $\eta_{t,k}$ are i.i.d. and independent, the overall corrupted gradients $W_{t}^{\prime }$ are i.i.d. and distributed as $N(0,\left(S_{\ell }\sigma_{w,t}^{2}+K\sigma^{2}\right)I)$, where $S_{\ell }=\sum_{k=1}^{K}S_{\ell ,k}$.

**Definition** **1** (Mutual information privacy [43]). *For every t∈{1,…,T}, if the mutual information $\frac{1}{q}I\left(W_{t};W_{t}^{\prime }\right)$ during the t-th round is upper bounded by ϵ, namely, $\max_{t\in \{1,\ldots ,T\}}\frac{1}{q}I\left(W_{t};W_{t}^{\prime }\right)\leq \epsilon$, the LDP mechanism is said to satisfy ϵ-mutual information privacy for ϵ>0.*

**Definition** **2** (Utility [44]). *The utility of $W_{t}^{\prime }$ is defined by the distortion between $W_{t}$ and $W_{t}^{\prime }$, and in this paper, we consider the quadratic distortion $d\left(W_{t},W_{t}^{\prime }\right)=\left|\right|W_{t}^{\prime }-W_{t}{\left|\right|}^{2}$. If $\frac{1}{qT}\sum_{t=1}^{T}E(d(W_{t},$ $W_{t}^{\prime }))\leq \upsilon$, the utility of $W_{t}^{\prime }$ is determined by υ, where the utility and the distortion have an inverse relationship with each other, i.e., smaller υ corresponds to larger utility.*

#### 2.2.2. Gradient Compression

We employ lossy Gaussian source coding characterized by a quadratic distortion metric, defined as $d\left(W_{t}^{\prime },{\hat{W}}_{t}^{\prime }\right)=\left|\right|W_{t}^{\prime }-{\hat{W}}_{t}^{\prime }{\left|\right|}^{2}$ (the source encoder and decoder are respectively located at the edge server and cloud server), where ${\hat{W}}_{t}^{\prime }$ is the output of the source decoder at the cloud server. Following [45] (Chapter 3.8, pp. 64–65), the edge server’s source encoder maps $W_{t}^{\prime }$ to $\left\{1,2,\ldots ,2^{qR_{t}\left(D\right)}\right\}$ and compresses $W_{t}^{\prime }$ into an index $W_{t}^{\prime \prime }$ that is uniformly distributed over $W_{t}^{\prime \prime }=\left\{1,2,\ldots ,2^{qR_{t}\left(D\right)}\right\}$. The rate-distortion function $R_{t}\left(D\right)$ is defined as follows:

(8) $$\begin{array}{c} R_{t}\left(D\right)=\left\{\begin{array}{cc} \frac{1}{2}\log\frac{K\sigma^{2}+S_{\ell }\sigma_{w,t}^{2}}{D} & 0\leq D<K\sigma^{2}+S_{\ell }\sigma_{w,t}^{2}\\ 0 & D\geq K\sigma^{2}+S_{\ell }\sigma_{w,t}^{2}, \end{array}\right. \end{array}$$

where $\frac{1}{qT}\sum_{t=1}^{T}E\left(d\left(W_{t}^{\prime },{\hat{W}}_{t}^{\prime }\right)\right)\leq D$. For the cloud server’s source decoder, the decoding mapping transforms the indices $\left\{1,2,\ldots ,2^{qR_{t}\left(D\right)}\right\}$ into ${\hat{W}}_{t}^{\prime }$. Here note that when $R_{t}\left(D\right)=0$, no message is transmitted, and ${\hat{W}}_{t}^{\prime }$ is set to 0.

#### 2.2.3. Communication Model

At the *t*-th round, the channel input-output relationships are expressed as follows:

(9) $$\begin{array}{c} Y_{i}\left(t\right)=hX_{i}\left(t\right)+\eta_{1,i}\left(t\right),\;\;1\leq i\leq N_{t}, \end{array}$$

(10) $$\begin{array}{c} {\tilde{Y}}_{i}\left(t\right)=\tilde{h}{\tilde{X}}_{i}\left(t\right)+\eta_{2,i}\left(t\right),\;\;1\leq i\leq N_{t}-1, \end{array}$$

(11) $$\begin{array}{c} Z_{i}\left(t\right)=gX_{i}\left(t\right)+\tilde{g}{\tilde{X}}_{i}\left(t\right)+\eta_{e,i}\left(t\right),\;\;1\leq i\leq N_{t}, \end{array}$$

where the input and output of the feedforward channel are denoted by $X_{i}\left(t\right)$ and $Y_{i}\left(t\right)$, respectively, the feedback channel’s input and output are ${\tilde{X}}_{i}\left(t\right)$ and ${\tilde{Y}}_{i}\left(t\right)$, respectively, and the eavesdropping channel’s output is $Z_{i}\left(t\right)$. Note that $X_{i}\left(t\right),{\tilde{Y}}_{i}\left(t\right)\in C^{A\times 1}$, ${\tilde{X}}_{i}\left(t\right),Y_{i}\left(t\right)\in C^{B\times 1}$ and $Z_{i}\left(t\right)\in C^{C\times 1}$. The average power constraint for the input of the edge server $X_{i}\left(t\right)$ is $\frac{1}{N_{t}}\sum_{i=1}^{N_{t}}E\left[X_{i}^{H}\left(t\right)X_{i}\left(t\right)\right]\leq P$, the input of the cloud server ${\tilde{X}}_{i}\left(t\right)$ is constrained by $\frac{1}{N_{t}-1}\sum_{i=1}^{N_{t}-1}E\left[{\tilde{X}}_{i}^{H}\left(t\right){\tilde{X}}_{i}\left(t\right)\right]\leq \tilde{P}$. The matrices $h\in C^{B\times A}$, $\tilde{h}\in C^{A\times B}$, $g\in C^{C\times A}$, and $\tilde{g}\in C^{C\times B}$ represent the CSI of the feedforward, feedback, and eavesdropping channels, respectively. The channel noises’ elements of $\eta_{e,i}\left(t\right)\in C^{C\times 1}$, $\eta_{2,i}\left(t\right)\in C^{A\times 1}$ and $\eta_{1,i}\left(t\right)\in C^{B\times 1}$ are i.i.d. and distributed as $\mathrm{CN}(0,\sigma_{e}^{2})$, $\mathrm{CN}(0,\sigma_{2}^{2})$ and $\mathrm{CN}(0,\sigma_{1}^{2})$, respectively. The input message $W_{t}^{\prime \prime }$ of the edge server is uniformly drawn in the set $W_{t}^{\prime \prime }$, and it is encoded as a codeword of length $N_{t}$. Furthermore, the input of the edge server is defined as $X_{i}\left(t\right)=f_{t,i}\left(W_{t}^{\prime \prime },h,\tilde{h},{\tilde{Y}}_{1}^{i-1}\left(t\right)\right)$, where $f_{t,i}\left(\cdot \right)$ is an encoding function and ${\tilde{Y}}_{1}^{i-1}\left(t\right)=\left({\tilde{Y}}_{1}\left(t\right),\ldots ,{\tilde{Y}}_{i-1}\left(t\right)\right)$. The cloud server estimates the message ${\hat{w}}_{t}^{\prime \prime }=\varphi \left(h,\tilde{h},Y^{N_{t}}\right)$ using the decoding function φ. The input of the cloud server is defined as ${\tilde{X}}_{i}\left(t\right)={\tilde{f}}_{t,i}\left(h,Y_{1}^{i}\left(t\right),\tilde{h}\right)$, where ${\tilde{f}}_{t,i}\left(\cdot \right)$ is an encoding function and $Y_{1}^{i}\left(t\right)=\left(Y_{1}\left(t\right),\ldots ,Y_{i}\left(t\right)\right)$. The average decoding error probability $P_{e,t}$ is given by the following:

(12) $$\begin{array}{c} P_{e,t}=\frac{1}{|W_{t}^{\prime \prime }|}\sum_{w_{t}^{\prime \prime }\in W_{t}^{\prime \prime }}Pr\left\{\varphi \left(h,Y^{N_{t}},\tilde{h}\right)\neq w_{t}^{\prime \prime }|w_{t}^{\prime \prime }\;\;sent\right\}. \end{array}$$

**Definition** **3.** *According to [46,47], the CSIs g and $\tilde{g}$ of eavesdropping channels are defined as follows:*

(13) $$\begin{array}{c} g=\hat{g}+{\Delta g,\;\;||\Delta g||}_{F}\leq \omega ,\;\;\;\tilde{g}=\hat{\tilde{g}}+\Delta \tilde{g},\;\;||\Delta \tilde{g}{\left|\right|}_{F}\leq \tilde{\omega }, \end{array}$$

*where $\hat{g}\in C^{C\times A},\hat{\tilde{g}}\in C^{C\times B}$ are the estimated CSI of g and $\tilde{g}$, respectively. Δg and $\Delta \tilde{g}$ represent the legal parties’ estimation errors about the perfect CSI of the eavesdropper’s channel, and these errors are respectively bounded by parameters ω>0 and $\tilde{\omega }>0$. Here note that $\Delta g=\Delta \tilde{g}=0$ corresponds to the situation that the legal parties obtain perfect CSI of the eavesdropper’s channel.*

**Definition** **4.** *The secrecy level of PLS [48] (the normalized uncertainty of the eavesdropper) is given by*

(14) $$\Delta =\frac{H(W_{1}^{\prime \prime },\ldots ,W_{T}^{\prime \prime }|Z^{N_{1}},\ldots ,Z^{N_{T}},h,\tilde{h},g,\tilde{g})}{H(W_{1}^{\prime \prime },\ldots ,W_{T}^{\prime \prime })},\;\;0\leq \Delta \leq 1.$$

*A transmission rate R is said to be (τ,N,δ,D,υ,ϵ) achievable, if for given decoding error probability τ, block length N ($N=\sum_{t=1}^{T}N_{t}$), secrecy level δ, $\frac{1}{qT}\sum_{t=1}^{T}E\left(d\left(W_{t}^{\prime },{\hat{W}}_{t}^{\prime }\right)\right)\leq D$, $\max_{t\in \{1,\ldots ,T\}}\frac{1}{q}I\left(W_{t};W_{t}^{\prime }\right)\leq \epsilon$ and $\frac{1}{qT}\sum_{t=1}^{T}E\left(d\left(W_{t},W_{t}^{\prime }\right)\right)\leq \upsilon$, there exists a channel code described above such that we have the following:*

(15) $$\frac{H(W_{1}^{\prime \prime },\ldots ,W_{T}^{\prime \prime })}{N}=R,\;\;\;\;\;\;\frac{1}{T}\sum_{t=1}^{T}P_{e,t}\leq \tau ,\;\;\;\;\;\;\Delta \geq \delta ,$$

*where δ∈[0,1], and δ=1 represents the perfect secrecy. For the WHFL in SISO/SIMO/MISO/MIMO cases, the achievable secrecy transmission rates are respectively denoted by $R_{siso/simo/miso/mimo}(\tau ,N,$δ,D,υ,ϵ), the channel gains are respectively defined by $h_{siso},{\tilde{h}}_{siso},g_{siso},{\tilde{g}}_{siso},h_{simo},{\tilde{h}}_{simo},g_{simo},$${\tilde{g}}_{simo},h_{miso},{\tilde{h}}_{miso},g_{miso},{\tilde{g}}_{miso}$, $h_{mimo},{\tilde{h}}_{mimo},g_{mimo}$, ${\tilde{g}}_{mimo}$, and the CSI estimation errors are defined by $\Delta g_{siso}$, $\Delta {\tilde{g}}_{siso}$, $\Delta g_{simo}$, $\Delta {\tilde{g}}_{simo}$, $\Delta g_{miso}$, $\Delta {\tilde{g}}_{miso}$, $\Delta g_{mimo}$ and $\Delta {\tilde{g}}_{mimo}$.*

### 2.3. Main Results

**Theorem** **1.** *For the MIMO WHFL with K users and T iterations, given that N, τ, υ, D, ϵ, δ, and applying the FBL approach in Section 3, the relationship between PLS, privacy, utility, and the noise variance of LDP is characterized by the following:*

(16) $$\max\left\{\underset{\mathrm{Secrecy}\mathrm{level}\mathrm{of}\mathrm{PLS}}{\underset{︸}{\max_{\begin{array}{c} t\in \{1,\ldots ,T\},\\ \Delta g_{mimo} \end{array}}\left[\frac{D\cdot 2^{\frac{2}{q(1-\delta )}\log\;\det\left(I+\frac{{\hat{g}}_{mimo}K_{x_{1}}{\hat{g}}_{mimo}^{H}}{\sigma_{e}^{2}}\right)}-S_{\ell }\sigma_{w,t}^{2}}{K}\right]}},\underset{\mathrm{Privacy}\mathrm{term}}{\underset{︸}{\max_{t\in \{1,\ldots ,T\}}\left[\frac{S_{\ell }\sigma_{w,t}^{2}}{K(2^{2\epsilon }-1)}\right]}}\right\}\leq \underset{\begin{array}{c} \mathrm{LDP}\mathrm{noise}\mathrm{variance} \end{array}}{\underset{︸}{\sigma^{2}}}\leq \underset{\begin{array}{c} \mathrm{Utility}\mathrm{term} \end{array}}{\underset{︸}{\frac{\upsilon }{K}}},$$

*where ${\hat{g}}_{mimo}=g_{mimo}-\Delta g_{mimo}$, $K_{x_{1}}=E\left(X_{1}\left(t\right)X_{1}^{H}\left(t\right)\right)$. In addition, an achievable transmission rate $R_{mimo}\left(\tau ,N,\delta ,D,\upsilon ,\epsilon \right)$ of our proposed FBL approach is given by the following:*

(17) $$R_{mimo}\left(\tau ,N,\delta ,D,\upsilon ,\epsilon \right)=\frac{\sum_{t=1}^{T}N_{t}R_{t}}{N},$$

*where*

(18) $$\begin{array}{c} N=\sum_{t=1}^{T}N_{t},\;\;\;R_{t}=\max_{\begin{array}{c} \sum_{j=1}^{J}P_{j}=P\\ \sum_{j=1}^{J}{\tilde{P}}_{j}=\tilde{P} \end{array}}\sum_{j=1}^{J}\frac{1}{N_{t}}\log\left(\frac{3{\mathrm{SNR}}_{j}d_{j}^{2}}{{\left[Q^{-1}\left(\frac{\tau }{8J}\right)\right]}^{2}}{\left(1+\frac{{\mathrm{SNR}}_{j}d_{j}^{2}}{\Psi_{1}\Psi_{2}}\right)}^{N_{t}-1}\right), \end{array}$$

(19) $$\begin{array}{c} \Psi_{1}=1+\xi \frac{d_{j}^{2}{\mathrm{SNR}}_{j}}{{\tilde{d}}_{j}^{2}{\tilde{\mathrm{SNR}}}_{j}},\;\;\Psi_{2}={\left(1-\frac{\xi }{{\tilde{d}}_{j}^{2}{\tilde{\mathrm{SNR}}}_{j}}\right)}^{-1},\;\;\xi =\frac{1}{3}{\left[Q^{-1}\left(\frac{\tau }{8J(N_{t}-1)}\right)\right]}^{2}, \end{array}$$

*and ${\mathrm{SNR}}_{j}=\frac{P_{j}}{\sigma_{1}^{2}}$, ${\tilde{\mathrm{SNR}}}_{j}=\frac{{\tilde{P}}_{j}}{\sigma_{2}^{2}}$, $d_{j}$, ${\tilde{d}}_{j}$, $P_{j}$, and ${\tilde{P}}_{j}$ are defined in Section 3.*

**Proof of Theorem 1.**  Our FBL approach for the MIMO WHFL is an extension of the classical SK scheme for the AWGN channel with noiseless feedback. The key to this extension is composed of three parts:

- The two-dimensional message mapping method, which maps the message to a complex codeword transmitted over the fading channels.
- An SVD-based pre-coding strategy that divides the MIMO channel into several parallel SISO channels.
- The two-dimensional modulo-lattice operation (MLO) that eliminates the impact of feedback channel noise on the performance of the SK scheme.

Details about the above tools and how to combine these tools to show our FBL approach for the MIMO WHFL are given in the next section, and the formal proof of Theorem 1 is in Appendix A. □

**Remark** **1.** *Here note that in the FBL approach for the MIMO WHFL, we apply an SVD-based pre-coding strategy to divide the MIMO channel into several parallel SISO channels, which indicates that the FBL approach for the SISO WHFL can be directly obtained since it is a special case of the approach for the MIMO WHFL. The following Corollary 1 proposes an FBL approach for the SISO WHFL and characterizes the relationship between PLS, privacy, utility, and the noise variance of LDP. Since Corollary 1 can be directly obtained from Theorem 1, we omit the detailed proof here.*

**Corollary** **1.** *For the SISO WHFL with K users and T iterations, given N, τ, υ, D, ϵ, δ, and using a similar FBL approach to that of Theorem 1, the relationship between PLS, privacy, utility, and the noise variance of LDP is characterized by the following:*

(20) $$\max\left\{\underset{\mathrm{Secrecy}\mathrm{level}\mathrm{of}\mathrm{PLS}}{\underset{︸}{\max_{\begin{array}{c} t\in \{1,\ldots ,T\},\\ \Delta g_{siso} \end{array}}\left[\frac{D\cdot 2^{\frac{2}{q(1-\delta )}\log\left(1+\frac{|{\hat{g}}_{siso}{|}^{2}P}{\sigma_{e}^{2}}\right)}-S_{\ell }\sigma_{w,t}^{2}}{K}\right]}},\underset{\mathrm{Privacy}\mathrm{term}}{\underset{︸}{\max_{t\in \{1,\ldots ,T\}}\left[\frac{S_{\ell }\sigma_{w,t}^{2}}{K(2^{2\epsilon }-1)}\right]}}\right\}\leq \underset{\begin{array}{c} \mathrm{LDP}\mathrm{noise}\mathrm{variance} \end{array}}{\underset{︸}{\sigma^{2}}}\leq \underset{\mathrm{Utility}\mathrm{term}}{\underset{︸}{\frac{\upsilon }{K}}},$$

*where ${\hat{g}}_{siso}=g_{siso}-\Delta g_{siso}$. Furthermore, an achievable transmission rate $R_{siso}\left(\tau ,N,\delta ,D,\upsilon ,\epsilon \right)$ of our proposed FBL approach is given by the following:*

(21) $$\begin{array}{c} R_{siso}\left(\tau ,N,\delta ,D,\upsilon ,\epsilon \right)=\frac{\sum_{t=1}^{T}N_{t}R_{t}}{N},\;\;R_{t}=\frac{1}{N_{t}}\log\left(\frac{3\mathrm{SNR}|h_{siso}{|}^{2}}{{\left[Q^{-1}\left(\frac{\tau }{8}\right)\right]}^{2}}{\left(1+\frac{\mathrm{SNR}|h_{siso}{|}^{2}}{\Psi_{3}\Psi_{4}}\right)}^{N_{t}-1}\right), \end{array}$$

*where $N=\sum_{t=1}^{T}N_{t}$, $\Psi_{3}=1+\xi^{★}\frac{|h_{siso}{|}^{2}\mathrm{SNR}}{|{\tilde{h}}_{siso}{|}^{2}\tilde{\mathrm{SNR}}}$, $\Psi_{4}={\left(1-\frac{\xi^{★}}{|{\tilde{h}}_{siso}{|}^{2}\tilde{\mathrm{SNR}}}\right)}^{-1}$, $\xi^{★}=\frac{1}{3}{\left[Q^{-1}\left(\frac{\tau }{8(N_{t}-1)}\right)\right]}^{2}$, $\mathrm{SNR}=\frac{P}{\sigma_{1}^{2}}$, $\tilde{\mathrm{SNR}}=\frac{\tilde{P}}{\sigma_{2}^{2}}$, and $|h_{siso}|$, $|{\tilde{h}}_{siso}|$, $|{\hat{g}}_{siso}|$ represent the modulus of $h_{siso}$, ${\tilde{h}}_{siso}$ and ${\hat{g}}_{siso}$, respectively.*

**Theorem** **2.** *For the SIMO WHFL with K users and T iterations, given N, τ, υ, D, ϵ, δ, and using the FBL approach in Section 4, the relationship between PLS, privacy, utility, and the noise variance of LDP is characterized by the following:*

(22) $$\begin{array}{c} \max\left\{\underset{\mathrm{Secrecy}\mathrm{level}\mathrm{of}\mathrm{PLS}}{\underset{︸}{\max_{\begin{array}{c} t\in \{1,\ldots ,T\},\\ \Delta g_{simo} \end{array}}\left[\frac{D\cdot 2^{\frac{2}{q(1-\delta )}\log\left(1+\frac{\left|\right|{\hat{g}}_{simo}{\left|\right|}^{2}P}{\sigma_{e}^{2}}\right)}-S_{\ell }\sigma_{w,t}^{2}}{K}\right]}},\underset{\mathrm{Privacy}\mathrm{term}}{\underset{︸}{\max_{t\in \{1,\ldots ,T\}}\left[\frac{S_{\ell }\sigma_{w,t}^{2}}{K(2^{2\epsilon }-1)}\right]}}\right\}\leq \underset{\begin{array}{c} \mathrm{LDP}\mathrm{noise}\mathrm{variance} \end{array}}{\underset{︸}{\sigma^{2}}}\leq \underset{\mathrm{Utility}\mathrm{term}}{\underset{︸}{\frac{\upsilon }{K}}}, \end{array}$$

*where ${\hat{g}}_{simo}=g_{simo}-\Delta g_{simo}$. In addition, an achievable transmission rate $R_{simo}\left(\tau ,N,\delta ,D,\upsilon ,\epsilon \right)$ of our proposed FBL approach is given by the following:*

(23) $$\begin{array}{c} R_{simo}\left(\tau ,N,\delta ,D,\upsilon ,\epsilon \right)=\frac{\sum_{t=1}^{T}N_{t}R_{t}}{N},\;\;R_{t}=\frac{1}{N_{t}}\log\left(\frac{3\mathrm{SNR}||h_{simo}{\left|\right|}^{2}}{{\left[Q^{-1}\left(\frac{\tau }{8}\right)\right]}^{2}}{\left(1+\frac{\mathrm{SNR}||h_{simo}{\left|\right|}^{2}}{\Psi_{5}\Psi_{6}}\right)}^{N_{t}-1}\right), \end{array}$$

*where $\Psi_{5}=1+\xi^{★}\frac{\left|\right|h_{simo}{\left|\right|}^{2}\mathrm{SNR}}{\left|\right|{\tilde{h}}_{simo}{\left|\right|}^{2}\tilde{\mathrm{SNR}}}$, $\Psi_{6}={\left(1-\frac{\xi^{★}}{\left|\right|{\tilde{h}}_{simo}{\left|\right|}^{2}\tilde{\mathrm{SNR}}}\right)}^{-1}$, SNR, $\tilde{\mathrm{SNR}}$, N, and $\xi^{★}$ are given in Corollary 1.*

**Proof of Theorem 2.**  The difference between the approaches in Theorems 1 and 2 is that for the SIMO case, we use a beamforming strategy together with a new pre-coding strategy instead of the SVD-based pre-coding strategy used for the MIMO case. Here the beamforming and new pre-coding strategies respectively transform the feedforward and feedback channels into SISO channels. Then, along the lines of the encoding-decoding procedure in Section 3.1.3, the FBL approach for the SIMO WHFL is obtained, and the detail about this approach is in Section 4. Finally, since the proof of Theorem 2 is included in that of Theorem 1, we omit the formal proof here. □

**Remark** **2.** *Here, note that in the SIMO WHFL, a beamforming strategy transforms the SIMO feedforward channel into the SISO feedforward channel, while a new pre-coding strategy transforms the MISO feedback channel into the SISO feedback channel. Analogously, for the MISO WHFL, first, we apply the pre-coding strategy of the SIMO WHFL to transform the MISO feedforward channel into the SISO feedforward channel, and the beamforming strategy of the SIMO WHFL to transform the SIMO feedback channel into the SISO feedback channel, then along the lines of the encoding-decoding procedure in Theorem 2, the following Corollary 2 for the MISO WHFL is obtained. As the proof follows a similar way to that of Theorem 2, the detailed proof is omitted here.*

**Corollary** **2.** *For the MISO WHFL with K users and T iterations, given N, τ, υ, D, ϵ and δ, and using a similar FBL approach to that of Theorem 2, the relationship between PLS, privacy, utility, and the noise variance of LDP is characterized by the following:*

(24) $$\begin{array}{c} \max\left\{\underset{\mathrm{Secrecy}\mathrm{level}\mathrm{of}\mathrm{PLS}}{\underset{︸}{\max_{\begin{array}{c} t\in \{1,\ldots ,T\},\\ \Delta g_{miso} \end{array}}\left[\frac{D\cdot 2^{\frac{2}{q(1-\delta )}\log\left(1+\frac{\left|\right|{\hat{g}}_{miso}{\left|\right|}^{2}P}{\sigma_{e}^{2}}\right)}-S_{\ell }\sigma_{w,t}^{2}}{K}\right]}},\underset{\mathrm{Privacy}\mathrm{term}}{\underset{︸}{\max_{t\in \{1,\ldots ,T\}}\left[\frac{S_{\ell }\sigma_{w,t}^{2}}{K(2^{2\epsilon }-1)}\right]}}\right\}\leq \underset{\begin{array}{c} \mathrm{LDP}\mathrm{noise}\mathrm{variance} \end{array}}{\underset{︸}{\sigma^{2}}}\leq \underset{\mathrm{Utility}\mathrm{term}}{\underset{︸}{\frac{\upsilon }{K}}}, \end{array}$$

*where ${\hat{g}}_{miso}=g_{miso}-\Delta g_{miso}$. In addition, an achievable transmission rate $R_{miso}\left(\tau ,N,\delta ,D,\upsilon ,\epsilon \right)$ of our proposed FBL approach is given by the following:*

(25) $$\begin{array}{c} R_{miso}\left(\tau ,N,\delta ,D,\upsilon ,\epsilon \right)=\frac{\sum_{t=1}^{T}N_{t}R_{t}}{N},\;\;R_{t}=\frac{1}{N_{t}}\log\left(\frac{3\mathrm{SNR}||h_{miso}{\left|\right|}^{2}}{{\left[Q^{-1}\left(\frac{\tau }{8}\right)\right]}^{2}}{\left(1+\frac{\mathrm{SNR}||h_{miso}{\left|\right|}^{2}}{\Psi_{7}\Psi_{8}}\right)}^{N_{t}-1}\right), \end{array}$$

*where $\Psi_{7}=1+\xi^{★}\frac{\left|\right|h_{miso}{\left|\right|}^{2}\mathrm{SNR}}{\left|\right|{\tilde{h}}_{miso}{\left|\right|}^{2}\tilde{\mathrm{SNR}}}$, $\Psi_{8}={\left(1-\frac{\xi^{★}}{\left|\right|{\tilde{h}}_{miso}{\left|\right|}^{2}\tilde{\mathrm{SNR}}}\right)}^{-1}$, SNR, $\tilde{\mathrm{SNR}}$, N and $\xi^{★}$ are given in Corollary 1.*

## 3. An FBL Approach for the MIMO WHFL

For the WHFL in the MIMO case, (9)–(11) can be re-written as follows:

(26) $$\begin{array}{c} Y_{i}\left(t\right)=h_{mimo}X_{i}\left(t\right)+\eta_{1,i}\left(t\right),\;\;1\leq i\leq N_{t}, \end{array}$$

(27) $$\begin{array}{c} {\tilde{Y}}_{i}\left(t\right)={\tilde{h}}_{mimo}{\tilde{X}}_{i}\left(t\right)+\eta_{2,i}\left(t\right),\;\;1\leq i\leq N_{t}-1, \end{array}$$

(28) $$\begin{array}{c} Z_{i}\left(t\right)=g_{mimo}X_{i}\left(t\right)+{\tilde{g}}_{mimo}{\tilde{X}}_{i}\left(t\right)+\eta_{e,i}\left(t\right),\;\;1\leq i\leq N_{t}, \end{array}$$

where $h_{mimo}\in C^{B\times A}$, ${\tilde{h}}_{mimo}\in C^{A\times B}$, $g_{mimo}\in C^{C\times A}$, ${\tilde{g}}_{mimo}\in C^{C\times B}$, $X_{i}\left(t\right)\in C^{A\times 1}$, ${\tilde{X}}_{i}\left(t\right)\in C^{B\times 1}$, the elements of $\eta_{1,i}\left(t\right)\in C^{B\times 1}$, $\eta_{2,i}\left(t\right)\in C^{A\times 1}$ and $\eta_{e,i}\left(t\right)\in C^{C\times 1}$ are i.i.d. as $\mathrm{CN}(0,\sigma_{1}^{2})$, $\mathrm{CN}(0,\sigma_{2}^{2})$ and $\mathrm{CN}(0,\sigma_{e}^{2})$, respectively. Here note that the feedforward channel (26) and the feedback channel (27) are both MIMO channels.

**Figure 3:**
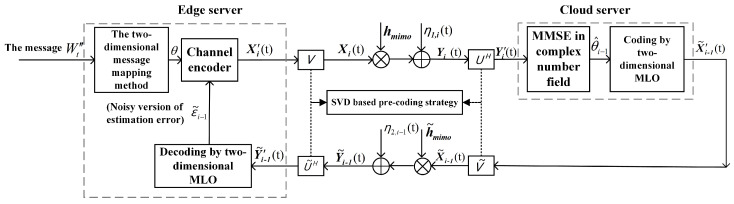
A schematic diagram of the FBL approach for the WHFL over the MIMO channel.

In this section, for the MIMO WHFL system, an FBL approach is proposed, which combines the two-dimensional message mapping method, the two-dimensional MLO, and the SVD technique, see the following Figure 3. To facilitate a better understanding of Figure 3, we introduce the two-dimensional message mapping method and the two-dimensional MLO below.

**Figure 4:**
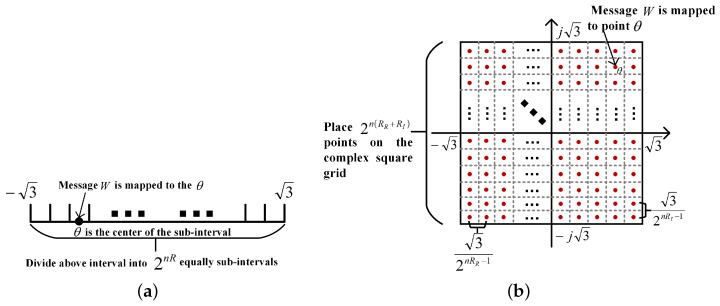
Comparison of the message mapping methods between the classical SK scheme and the scheme in this paper. (**a**) Message mapping of classical SK scheme. (**b**) Message mapping in this paper.

**The two-dimensional message mapping method**: We first review the message mapping in the classical SK scheme [35] (see Figure 4a). Specifically, for given codeword length *n*, let the message $W\in W=\{1,2,\ldots ,2^{nR}\}$ and $\left|W\right|=2^{nR}$, where *R* is the transmission rate. Partition the interval $\left[-\sqrt{3},\sqrt{3}\right]$ into $2^{nR}$ equal sub-intervals, with each sub-interval’s midpoint corresponding to a message in W. Let θ denote the midpoint associated with message *W*, where the variance of θ is approximately 1. This one-dimensional mapping method is shown to be optimal for AWGN channels with real signals. To address the complexity of fading channels, we introduce a two-dimensional message mapping method, detailed as follows:

For given codeword length *n*, let message $W=(W_{R},W_{I})$, where *W*, $W_{R}$ and $W_{I}$ are uniformly distributed in $W=\{1,\ldots ,2^{nR}\}$, $W_{R}=\left\{1,\ldots ,2^{nR_{R}}\right\}$ and $W_{I}=\left\{1,\ldots ,2^{nR_{I}}\right\}$, respectively, and $R_{R}+R_{I}=R$. Since the message *W* is composed of two parts, we place the points $\left(W_{R},W_{I}\right)$ in a complex square grid with corners located at $\left(\pm \sqrt{3},\pm j\sqrt{3}\right)$ (see Figure 4b). Divide the entire square grid into $2^{n(R_{R}+R_{I})}$ equally spaced sub-grids, and the center point of each sub-grid is mapped to a pair of values in $W=(W_{R},W_{I})$. Let $\theta =\theta_{R}+j\theta_{I}$ be the center point of the sub-grid with respect to (w.r.t) the message $W=(W_{R},W_{I})$, where $\theta_{R}$ and $\theta_{I}$ represent the real and imaginary components of θ, respectively, and the variance of θ approximately equals 2.

**The two-dimensional MLO**: The two-dimensional MLO is given by the following:

(29) $$\begin{array}{c} M_{\Lambda }\left[x\right]\overset{\mathrm{def}}{=}x-Q\left[x\right], \end{array}$$

where the two-dimensional lattice $\Lambda =\Lambda_{R}+j\Lambda_{I}$ is a complex plane with $\Lambda_{R}\in \left[-\frac{d}{2},-\frac{d}{2}\right]$, $\Lambda_{I}\in \left[-\frac{d}{2},-\frac{d}{2}\right]$, d>0, $j=\sqrt{-1}$, Q[x] is the nearest neighbor quantization of *x* w.r.t. Λ, and *x* is a complex-valued number. Some basic properties of the two-dimensional MLO [39] are listed below.

**Proposition 1.**  *(1). The distributive law $M_{\Lambda }\left[M_{\Lambda }\left[x\right]+y\right]=M_{\Lambda }\left[x+y\right]$.*

*(2). If x+y∈Λ, $M_{\Lambda }\left[x+y\right]=x+y$, otherwise, a* modulo-aliasing error *occurred.*

*(3). Let the dither signal ν be uniformly distributed on* Λ*, then $M_{\Lambda }\left[x+\nu \right]$ is uniformly distributed on* Λ*, where $\mathrm{Var}\left(M_{\Lambda }\left[x+\nu \right]\right)=\frac{d^{2}}{12}+\frac{d^{2}}{12}=\frac{d^{2}}{6}$.*

**Figure 5:**
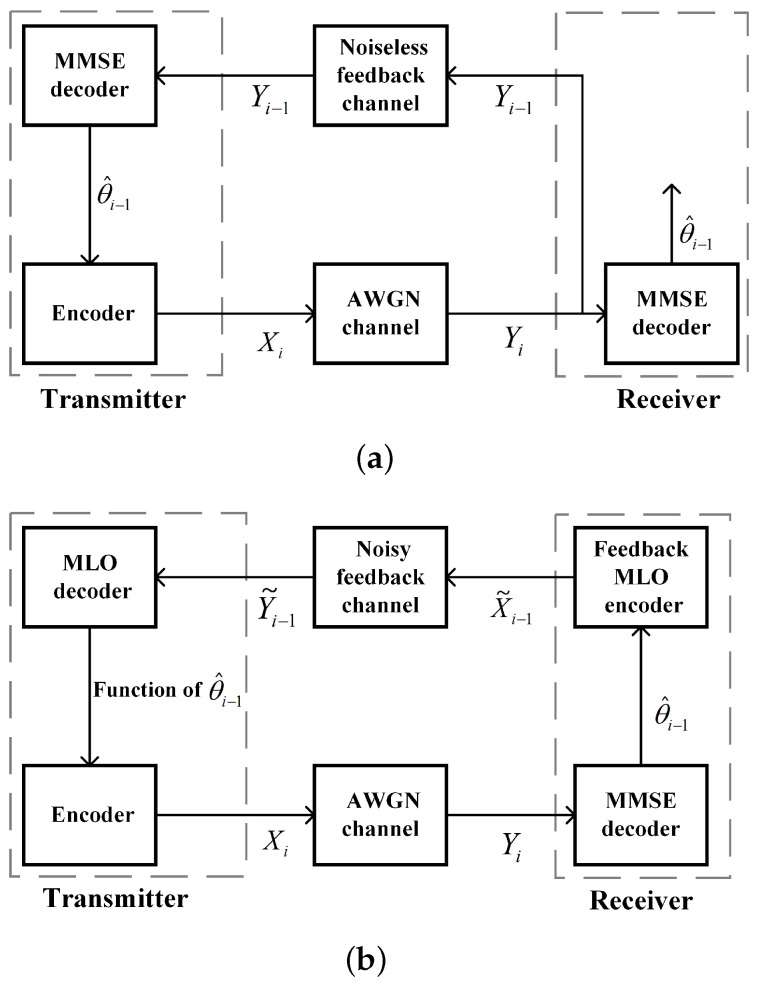
Comparing the mechanisms between the classical SK scheme and the two-dimensional MLO-based SK-type scheme, where ${\hat{\theta }}_{i-1}$ represents the estimation of the transmitted message θ at time i−1. (**a**) The classical SK scheme in a certain round *i*. (**b**) The two-dimensional MLO-based SK-type scheme in a certain round *i*.

The classical SK scheme does not work in the noisy feedback case, and this is because in such a case, the transmitter cannot accurately obtain the estimation error of the receiver. We show that by applying the two-dimensional MLO to both the feedforward and feedback encoders, the adverse effects of feedback channel noise on the SK scheme’s performance can be mitigated, which allows the SK-type scheme to remain effective even in the presence of noisy feedback. The following Figure 5a,b illustrate the differences between the classical SK scheme and the modified SK-type scheme utilizing two-dimensional MLO.

### 3.1. An FBL Approach for the MIMO WHFL

#### 3.1.1. Channel Decomposition by SVD

Based on the SVD technique, matrices $h_{mimo}$ and ${\tilde{h}}_{mimo}$ can be expressed as follows:

(30) $$\begin{array}{c} h_{mimo}=U\Lambda V^{H},\;\;{\tilde{h}}_{mimo}=\tilde{U}\tilde{\Lambda }{\tilde{V}}^{H}, \end{array}$$

where $U,{\tilde{V}}^{H}\in C^{B\times B}$ and $\tilde{U},V^{H}\in C^{A\times A}$ are unitary matrices. The diagonal matrices $\Lambda \in C^{B\times A}$ and $\tilde{\Lambda }\in C^{A\times B}$ have non-negative real number diagonal elements ($d_{1}$,…,$d_{J}$) and (${\tilde{d}}_{1}$,…,${\tilde{d}}_{J}$) [49], respectively, and

(31) J=min(A,B).

According to (26) and (30), we have the following:

(32) $$U^{H}Y_{i}\left(t\right)=\Lambda V^{H}X_{i}\left(t\right)+U^{H}\eta_{1,i}\left(t\right)⟹Y_{i}^{\prime }\left(t\right)=\Lambda X_{i}^{\prime }\left(t\right)+\eta_{1,i}^{\prime }\left(t\right),$$

where $Y_{i}^{\prime }\left(t\right)=U^{H}Y_{i}\left(t\right),\eta_{1,i}^{\prime }\left(t\right)=U^{H}\eta_{1,i}\left(t\right)\in C^{B\times 1}$ and $X_{i}^{\prime }\left(t\right)=V^{H}X_{i}\left(t\right)\in C^{A\times 1}$. It is noted that $E\left(X_{i}^{\prime H}\left(t\right)X_{i}^{\prime }\left(t\right)\right)=E\left(X_{i}^{H}\left(t\right)X_{i}\left(t\right)\right)$ and $E\left(\eta_{1,i}^{\prime H}\left(t\right)\eta_{1,i}^{\prime }\left(t\right)\right)=E\left(\eta_{1,i}^{H}\left(t\right)\eta_{1,i}\left(t\right)\right)$, ensuring that the power constraint of $X_{i}\left(t\right)$ is equal to that of $X_{i}^{\prime }\left(t\right)$, and the distributions of $\eta_{1,i}^{\prime }\left(t\right)$ and $\eta_{1,i}\left(t\right)$ remain the same. As Λ is a diagonal matrix, (32) can be decomposed as follows:

(33) $$Y_{j,i}^{\prime }\left(t\right)=d_{j}X_{j,i}^{\prime }\left(t\right)+\eta_{j,1,i}^{\prime }\left(t\right),\;\;1\leq j\leq J,\;\;1\leq i\leq N_{t},$$

where $Y_{j,i}^{\prime }\left(t\right)$, $X_{j,i}^{\prime }\left(t\right)$ and $\eta_{j,1,i}^{\prime }\left(t\right)$ denote the *j*-th components of $Y_{i}^{\prime }\left(t\right)$, $X_{i}^{\prime }\left(t\right)$ and $\eta_{1,i}^{\prime }\left(t\right)$, respectively.

Similarly, from (27) and (30), (27) can be decomposed as follows:

(34) $${\tilde{Y}}_{j,i}^{\prime }\left(t\right)={\tilde{d}}_{j}{\tilde{X}}_{j,i}^{\prime }\left(t\right)+\eta_{j,2,i}^{\prime }\left(t\right),\;\;1\leq j\leq J,\;\;1\leq i\leq N_{t}-1,$$

where ${\tilde{Y}}_{j,i}^{\prime }\left(t\right)$, ${\tilde{X}}_{j,i}^{\prime }\left(t\right)$ and $\eta_{j,2,i}^{\prime }\left(t\right)$ denote the *j*-th components of ${\tilde{Y}}_{i}^{\prime }\left(t\right)$, ${\tilde{X}}_{i}^{\prime }\left(t\right)$ and $\eta_{2,i}^{\prime }\left(t\right)$, respectively, and ${\tilde{Y}}_{i}^{\prime }\left(t\right)={\tilde{U}}^{H}{\tilde{Y}}_{i}\left(t\right),\eta_{2,i}^{\prime }\left(t\right)={\tilde{U}}^{H}\eta_{2,i}\left(t\right)\in C^{A\times 1}$ and ${\tilde{X}}_{i}^{\prime }\left(t\right)={\tilde{V}}^{H}{\tilde{X}}_{i}\left(t\right)\in C^{B\times 1}$. As shown in (32)–(34), applying the SVD technique, the feedforward and feedback MIMO channels can be effectively transformed into *J* parallel SISO sub-channels.

*Power allocating*: The edge server assigns power $P_{1},\ldots ,P_{J}$ to the *J* parallel sub-channels for the feedforward channel, where $\sum_{j=1}^{J}P_{j}=P$. Similarly, the cloud server distributes power ${\tilde{P}}_{1},\ldots ,{\tilde{P}}_{J}$ across the *J* parallel sub-channels for the feedback channel, where $\sum_{j=1}^{J}{\tilde{P}}_{j}=\tilde{P}$.

#### 3.1.2. Message Splitting

For given τ, $N_{t}$, υ, *D* and ϵ, we define the following:

(35) $$\begin{array}{c} |W_{t}^{\prime \prime }|=2^{N_{t}R_{t}}=2^{qR_{t}\left(D\right)},\;\;R_{t}=\frac{H(W_{t}^{\prime \prime })}{N_{t}}. \end{array}$$

Next, the message $W_{t}^{\prime \prime }$ is divided into *J* independent components $\left(W_{t,1}^{\prime \prime },\ldots ,W_{t,J}^{\prime \prime }\right)$, where $W_{t,j}^{\prime \prime }$ is uniformly distributed over the set $W_{t,j}^{\prime \prime }=\left\{1,2,\ldots ,2^{N_{t}R_{t,j}}\right\}$ and j=1,…,J. Then, we divide each sub-message $W_{t,j}^{\prime \prime }$ into $W_{t,j}^{\prime \prime }=\left(W_{t,j,R}^{\prime \prime },W_{t,j,I}^{\prime \prime }\right)$, where $W_{t,j,R}^{\prime \prime }$ and $W_{t,j,I}^{\prime \prime }$ are uniformly distributed over the sets $W_{t,j,R}^{\prime \prime }=\left\{1,2,\ldots ,2^{N_{t}R_{t,j,R}}\right\}$ and $W_{t,j,I}^{\prime \prime }=\left\{1,2,\ldots ,2^{N_{t}R_{t,j,I}}\right\}$, respectively. The rate for each parallel sub-channel is defined as $R_{t,j}=R_{t,j,R}+R_{t,j,I}$. Consequently, the total rate $R_{t}$ for all *J* parallel sub-channels during the *t*-th communication round is as follows:

(36) $$R_{t}=\sum_{j=1}^{J}\left(R_{t,j,R}+R_{t,j,I}\right).$$

#### 3.1.3. An FBL Scheme of Each Parallel Sub-Channel

By using the two-dimensional message mapping method introduced in the last subsection, the message $W_{t,j}^{\prime \prime }$ is mapped to the center point $\theta_{j}$ of its corresponding sub-grid.

**Initialization**: At time instant 1, the edge server maps the messages $W_{t,j}^{\prime \prime }$ to $\theta_{j}=\theta_{R,j}+j\theta_{I,j}$, and sends the following:

(37) $$\begin{array}{c} X_{j,1}^{\prime }\left(t\right)=\sqrt{\frac{P_{j}}{2}}\theta_{j}, \end{array}$$

Then, the cloud server computes the first estimation ${\hat{\theta }}_{j,1}$ of $\theta_{j}$ by the following:

(38) $$\begin{array}{c} {\hat{\theta }}_{j,1}=\frac{Y_{j,1}^{\prime }\left(t\right)}{d_{j}\sqrt{\frac{P_{j}}{2}}}=\theta_{j}+\frac{\eta_{j,1,1}^{\prime }\left(t\right)}{d_{j}\sqrt{\frac{P_{j}}{2}}}=\theta_{j}+\varepsilon_{1}, \end{array}$$

where $\varepsilon_{1}=\varepsilon_{R,1}+j\varepsilon_{I,1}={\hat{\theta }}_{j,1}-\theta_{j}$ is the estimation error of the cloud server at time instant 1. Define $\alpha_{1}=\mathrm{Var}\left(\varepsilon_{1}\right)=\frac{2\sigma_{1}^{2}}{d_{j}^{2}P_{j}}$, $\alpha_{R,1}=\mathrm{Var}\left(\varepsilon_{R,1}\right)=\frac{\sigma_{1}^{2}}{d_{j}^{2}P_{j}}$ and $\alpha_{I,1}=\mathrm{Var}\left(\varepsilon_{I,1}\right)=\frac{\sigma_{1}^{2}}{d_{j}^{2}P_{j}}$.

**Iteration**: First, we introduce a shared dither random i.i.d. sequence $\nu^{N_{t}-1}=\left(\nu_{1},\ldots ,\nu_{N_{t}-1}\right)$, which is perfectly known by both the edge server and the cloud server, and it is uniformly distributed on Λ ($\Lambda =\Lambda_{R}+j\Lambda_{I}$ is a complex plane with $\Lambda_{R}\in \left[-\frac{d}{2},-\frac{d}{2}\right]$, $\Lambda_{I}\in \left[-\frac{d}{2},-\frac{d}{2}\right]$), and $d=\sqrt{6{\tilde{P}}_{j}}$. Here $\nu^{N_{t}-1}$ is independent of all signals transmitted over channels. At time instant *i* ($2\leq i\leq N_{t}$), using the two-dimensional MLO shown in Section 3, the cloud server sends the following:

(39) $$\begin{array}{c} {\tilde{X}}_{j,i-1}^{\prime }\left(t\right)=M_{\Lambda }\left[\gamma_{i-1}{\hat{\theta }}_{j,i-1}+\nu_{i-1}\right], \end{array}$$

where $\gamma_{i-1}$ is a modulation coefficient. From Property (3) of Proposition 1, we have $E\left({\tilde{X}}_{j,i-1}^{\prime H}\left(t\right){\tilde{X}}_{j,i-1}^{\prime }\left(t\right)\right)={\tilde{P}}_{j}$ (the dither signals guarantee that the codeword transmitted by the cloud server meets the power constraint). Then the edge server computes a noisy version of estimation error $\varepsilon_{i-1}={\hat{\theta }}_{j,i-1}-\theta_{j}$ by the following:

(40) $$\begin{array}{c} {\tilde{\varepsilon }}_{i-1}=\frac{1}{\gamma_{i-1}}M_{\Lambda }\left[\frac{{\tilde{Y}}_{j,i-1}^{\prime }\left(t\right)}{{\tilde{d}}_{j}}-\gamma_{i-1}\theta_{j}-\nu_{i-1}\right]\overset{\left(a\right)}{=}\frac{1}{\gamma_{i-1}}M_{\Lambda }\left[\gamma_{i-1}\varepsilon_{i-1}+\frac{\eta_{j,2,i-1}^{\prime }\left(t\right)}{{\tilde{d}}_{j}}\right], \end{array}$$

where (a) is due to the modulo distributive law in property (1) of Proposition 1. The *modulo-aliasing errors* do not occur in the edge server, if $\gamma_{i-1}\varepsilon_{i-1}+\frac{\eta_{j,2,i-1}^{\prime }\left(t\right)}{{\tilde{d}}_{j}}\in \Lambda$. Hence, the edge server obtains ${\tilde{\varepsilon }}_{i-1}=\varepsilon_{i-1}+\frac{\eta_{j,2,i-1}^{\prime }\left(t\right)}{\gamma_{i-1}{\tilde{d}}_{j}}$. Then, the edge server sends the following:

(41) $$\begin{array}{c} X_{j,i}^{\prime }\left(t\right)=\lambda_{i-1}\gamma_{i-1}{\tilde{\varepsilon }}_{i-1}, \end{array}$$

where $\lambda_{i-1}$ is chosen to satisfy the transmitter’s power constraint $P_{j}$. Then, the cloud server updates ${\hat{\theta }}_{j,i}$ by computing the following:

(42) $$\begin{array}{c} {\hat{\theta }}_{j,i}={\hat{\theta }}_{j,i-1}-{\hat{\varepsilon }}_{i-1}={\hat{\theta }}_{j,i-1}-\beta_{i}\frac{Y_{j,i}^{\prime }\left(t\right)}{d_{j}}, \end{array}$$

where ${\hat{\varepsilon }}_{i-1}=\beta_{i}\frac{Y_{j,i}^{\prime }\left(t\right)}{d_{j}}$, and the MMSE estimation coefficient $\beta_{i}$ is given by the following:

(43) $$\begin{array}{c} \beta_{i}=\frac{E(\frac{\varepsilon_{i-1}Y_{j,i}^{\prime }{\left(t\right)}^{H}}{d_{j}})}{E(\frac{Y_{j,i}^{\prime }\left(t\right)Y_{j,i}^{\prime }{\left(t\right)}^{H}}{d_{j}^{2}})}, \end{array}$$

which ensures that $\varepsilon_{i-1}$ is correctly estimated from $Y_{j,i}^{\prime }\left(t\right)$. Define $\varepsilon_{i}=\varepsilon_{R,i}+j\varepsilon_{I,i}={\hat{\theta }}_{j,i}-\theta_{j}$, (42) yields the following:

(44) $$\begin{array}{c} \varepsilon_{i}=\varepsilon_{i-1}-\beta_{i}\frac{Y_{j,i}^{\prime }\left(t\right)}{d_{j}}. \end{array}$$

Further define $\alpha_{i}=\mathrm{Var}\left(\varepsilon_{i}\right)$, $\alpha_{R,i}=\mathrm{Var}\left(\varepsilon_{R,i}\right)$, $\alpha_{I,i}=\mathrm{Var}\left(\varepsilon_{I,i}\right)$. Since $\varepsilon_{i}$ is a CSCG distribution estimation error, we conclude that $\alpha_{R,i}=\alpha_{I,i}=\frac{\alpha_{i}}{2}$.

**Decoding**: At time instant $N_{t}$, the final estimation obtained by the cloud server is ${\hat{\theta }}_{j,N_{t}}=\theta_{j}+\varepsilon_{N_{t}}$, where $\varepsilon_{N_{t}}=\varepsilon_{R,N_{t}}+j\varepsilon_{I,N_{t}}$. The cloud server successfully decodes the message $W_{t,j}^{\prime \prime }$ if ${\hat{\theta }}_{j,N_{t}}$ is closest to the message point $\theta_{j}$, i.e., $\varepsilon_{R,N_{t}}\in \left[-\frac{\sqrt{3}}{2^{N_{t}R_{t,j,R}}},\frac{\sqrt{3}}{2^{N_{t}R_{t,j,R}}}\right)$ and $\varepsilon_{I,N_{t}}\in \left[-\frac{\sqrt{3}}{2^{N_{t}R_{t,j,I}}},\frac{\sqrt{3}}{2^{N_{t}R_{t,j,I}}}\right)$.

The formal proof of Theorem 1 is provided in Appendix A.

## 4. An FBL Approach for the SIMO WHFL

**Figure 6:**
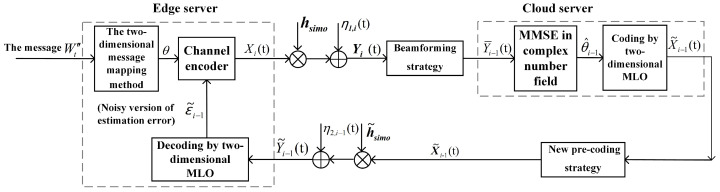
A schematic diagram of the FBL approach for the SIMO WHFL.

For the SIMO WHFL, (9)–(11) can be re-written as follows:

(45) $$\begin{array}{c} Y_{i}\left(t\right)=h_{simo}X_{i}\left(t\right)+\eta_{1,i}\left(t\right),\;\;1\leq i\leq N_{t}, \end{array}$$

(46) $$\begin{array}{c} {\tilde{Y}}_{i}\left(t\right)={\tilde{h}}_{simo}{\tilde{X}}_{i}\left(t\right)+\eta_{2,i}\left(t\right),\;\;1\leq i\leq N_{t}-1, \end{array}$$

(47) $$\begin{array}{c} Z_{i}\left(t\right)=g_{simo}X_{i}\left(t\right)+{\tilde{g}}_{simo}{\tilde{X}}_{i}\left(t\right)+\eta_{e,i}\left(t\right),\;\;1\leq i\leq N_{t}, \end{array}$$

where $h_{simo}\in C^{B\times 1}$, ${\tilde{h}}_{simo}\in C^{1\times B}$, $g_{simo}\in C^{C\times 1}$, ${\tilde{g}}_{simo}\in C^{C\times B}$, $X_{i}\left(t\right)\in C^{1\times 1}$, ${\tilde{X}}_{i}\left(t\right)\in C^{B\times 1}$, the elements of $\eta_{1,i}\left(t\right)\in C^{B\times 1}$ and $\eta_{e,i}\left(t\right)\in C^{C\times 1}$ are i.i.d. as $\mathrm{CN}(0,\sigma_{1}^{2})$ and $\mathrm{CN}(0,\sigma_{e}^{2})$, respectively, and $\eta_{2,i}\left(t\right)\in C^{1\times 1}∼\mathrm{CN}\left(0,\sigma_{2}^{2}\right)$. Here note that the feedforward channel (45) is a SIMO channel, while the feedback channel (46) is a MISO channel. Unlike the SVD technique used for the MIMO WHFL that decomposes the MIMO channel into several parallel SISO channels, we use a beamforming strategy to transform the feedforward SIMO channel into the SISO channel, and a new pre-coding strategy to transform the feedback MISO channel into the SISO channel, see the following Figure 6. Further applying the approach for each SISO channel (see Section 3.1.3), the FBL approach for the SIMO WHFL is obtained, and the details are given below.

*Beamforming strategy:* The signal received by the cloud server in (45) can proceed as follows:

(48) $$\begin{array}{c} h_{simo}^{H}Y_{i}\left(t\right)=h_{simo}^{H}h_{simo}X_{i}\left(t\right)+h_{simo}^{H}\eta_{1,i}\left(t\right)=\left|\right|h_{simo}{\left|\right|}^{2}X_{i}\left(t\right)+h_{simo}^{H}\eta_{1,i}\left(t\right),\\ ⟹{\bar{Y}}_{i}\left(t\right)=\left|\right|h_{simo}{\left|\right|}^{2}X_{i}\left(t\right)+{\bar{\eta }}_{1,i}\left(t\right), \end{array}$$

where ${\bar{Y}}_{i}\left(t\right)=h_{simo}^{H}Y_{i}\left(t\right)\in C^{1\times 1}$ and ${\bar{\eta }}_{1,i}\left(t\right)=h_{simo}^{H}\eta_{1,i}\left(t\right)\in C^{1\times 1}$. Applying (4), the feedforward SIMO channel is transformed into the SISO channel.

*A new pre-coding strategy:* For the feedback channel (46), allowing the following:

(49) $$\begin{array}{c} {\tilde{X}}_{i}\left(t\right)=\frac{{\tilde{h}}_{simo}^{H}}{\left|\right|{\tilde{h}}_{simo}\left|\right|}{\tilde{X}}_{i}\left(t\right), \end{array}$$

where ${\tilde{X}}_{i}\left(t\right)\in C^{1\times 1}$ and $E\left({\tilde{X}}_{i}^{H}\left(t\right){\tilde{X}}_{i}\left(t\right)\right)=E\left({\tilde{X}}_{i}^{H}\left(t\right)\frac{{\tilde{h}}_{simo}}{\left|\right|{\tilde{h}}_{simo}\left|\right|}\frac{{\tilde{h}}_{simo}^{H}}{\left|\right|{\tilde{h}}_{simo}\left|\right|}{\tilde{X}}_{i}\left(t\right)\right)=E\left({\tilde{X}}_{i}^{H}\left(t\right){\tilde{X}}_{i}\left(t\right)\right)=\tilde{P}$, which indicates that the power constraint of ${\tilde{X}}_{i}\left(t\right)$ is equal to that of ${\tilde{X}}_{i}\left(t\right)$. Hence, substituting (49) into (46), we have the following:

(50) $$\begin{array}{c} {\tilde{Y}}_{i}\left(t\right)={\tilde{h}}_{simo}\frac{{\tilde{h}}_{simo}^{H}}{\left|\right|{\tilde{h}}_{simo}\left|\right|}{\tilde{X}}_{i}\left(t\right)+\eta_{2,i}\left(t\right)=\left|\right|{\tilde{h}}_{simo}\left|\right|{\tilde{X}}_{i}\left(t\right)+\eta_{2,i}\left(t\right), \end{array}$$

which indicates that the feedback MISO channel is transformed into the SISO channel. Hence along the lines of the encoding-decoding procedure in Section 3.1.3, the FBL approach for the SIMO WHFL is obtained.

Since the proof of Theorem 2 is included in the proof of Theorem 1, we omit the detailed proof here.

## 5. Simulation Results

### 5.1. Experimental Settings

The simulation results are derived by averaging 2000 independent channel realizations (i.e., Monte-Carlo simulations). We consider a WHFL system consisting of 10 users, an edge server, and a cloud server, with each user having the same amount of training data. We assume that the channel matrix elements follow an i.i.d. distribution as CN(0,1) [5,6,17]. Following [47], the maximum normalized estimation errors of the eavesdropper’s channel are defined as $\Omega =\frac{\omega }{{\left||g|\right|}_{F}}$ and $\tilde{\Omega }=\frac{\tilde{\omega }}{\left|\right|\tilde{g}{\left|\right|}_{F}}$, where ω and $\tilde{\omega }$ are defined in (13). The edge server employs Lempel–Ziv–Welch (LZW) source coding [50] to compress the quantized gradients, and the total transmitted data are *M* bits. The transmission latency for the edge server to upload data is $T_{comm}=\frac{M}{R_{eg}}$ [5], where $R_{eg}$ represents the edge server’s transmission rate.

To evaluate the effectiveness of the proposed FBL scheme under real-world conditions, we train a neural network using the MNIST dataset (http://yann.lecun.com/exdb/mnist/, accessed on 20 March 2024), which contains 60,000 training samples and 10,000 test samples of 10 different handwritten digits. The network architecture includes 784 input nodes, a hidden layer containing 20 nodes, and an output layer with 10 nodes. The loss function is cross-entropy, with the hidden and output layers utilizing the ReLU and softmax activation functions, respectively. The neural network contains a total of q=15,910 parameters, and the learning rate is set at μ=0.1. In the experiments, the following three schemes are compared.

- Benchmark (Perfect HFL): The perfectly aggregated HFL system can be achieved through error-free transmission, which serves as the benchmark accuracy in ideal settings.
- Baseline 1 (Random binning coding scheme (RBCS)-based WHFL [26,28]): The gradient data from the edge servers is uploaded using the RBCS, which is based on traditional low-density parity-check (LDPC) codes with a target bit error rate of $10^{-6}$.
- Baseline 2 (Frequency division multiple access (FDMA)-based WHFL with artificial noise (AN) [29]): In the FDMA-based WHFL system with AN, FDMA is employed to transmit gradient data from edge servers to the cloud server, targeting a bit error ratio of $10^{-6}$. Additionally, AN is added to the transmitted signals to prevent eavesdroppers from obtaining the true gradient data.

### 5.2. Experimental Results

**Figure 7:**
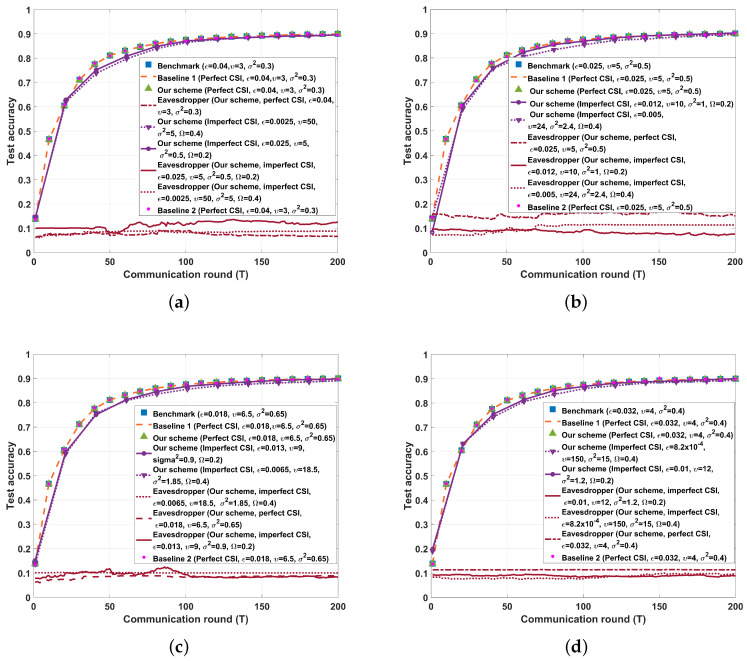
Performance comparison between the different schemes on the MNIST dataset (K=10, $S_{\ell }=60,000$, q=15,910, $D=10^{-4}$, $\tilde{\mathrm{SNR}}=15$ dB, P=10, $\tau =10^{-6}$, $\sigma_{1}^{2}=\sigma_{2}^{2}=1,\sigma_{e}^{2}=2$). (**a**) A=B=C=4, δ=0.99994. (**b**) A=1, B=C=4, δ=0.99997. (**c**) A=4, B=C=1, δ=0.99997. (**d**) A=B=C=1, δ=0.99998.

**Figure 8:**
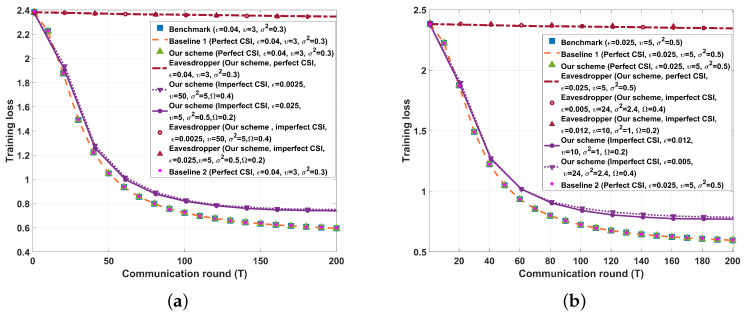

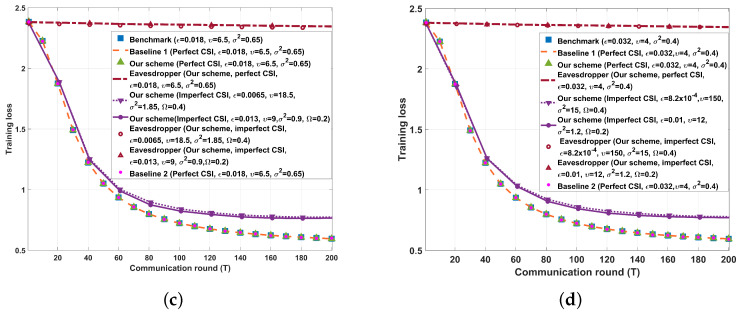
Performance comparison between the different schemes on the MNIST dataset (K=10, $S_{\ell }=60,000$, q=15,910, $D=10^{-4}$, $\tilde{\mathrm{SNR}}=15$ dB, P=10, $\tau =10^{-6}$, $\sigma_{1}^{2}=\sigma_{2}^{2}=1,\;\sigma_{e}^{2}=2$). (**a**) A=B=C=4, δ=0.99994. (**b**) A=1, B=C=4, δ=0.99997. (**c**) A=4, B=C=1, δ=0.99997. (**d**) A=B=C=1, δ=0.99998.

We show the results of test accuracy and the cross entropy versus the communication round for SISO/SIMO/MISO/MIMO cases in Figure 7 and Figure 8, respectively. From Figure 7 and Figure 8, we see that if perfect CSI of the eavesdropper’s channel is obtained by legal parties, both our proposed FBL scheme, Baseline 1 scheme, and Baseline 2 scheme almost do not affect the learning performance of HFL. This is because Baseline 1, Baseline 2, and our proposed schemes are all capable of transmitting gradient data with a sufficiently low decoding error probability. On the other hand, in our proposed FBL schemes, if imperfect CSI of the eavesdropper’s channel is obtained by legal parties, the test accuracy of HFL decreases, and the training loss of HFL increases as the maximum normalized estimation error of the eavesdropper’s channel increases. However, note that in such an imperfect CSI case, our proposed FBL schemes still provide the same level of secrecy as that of the perfect CSI case, which shows the robustness of our schemes against imperfect CSI of the eavesdropper’s channel. Furthermore, Figure 7 and Figure 8 demonstrate that the eavesdropper cannot obtain the real gradient data when applying our FBL scheme, which indicates that our FBL schemes effectively ensure the PLS of the data.

**Figure 9:**
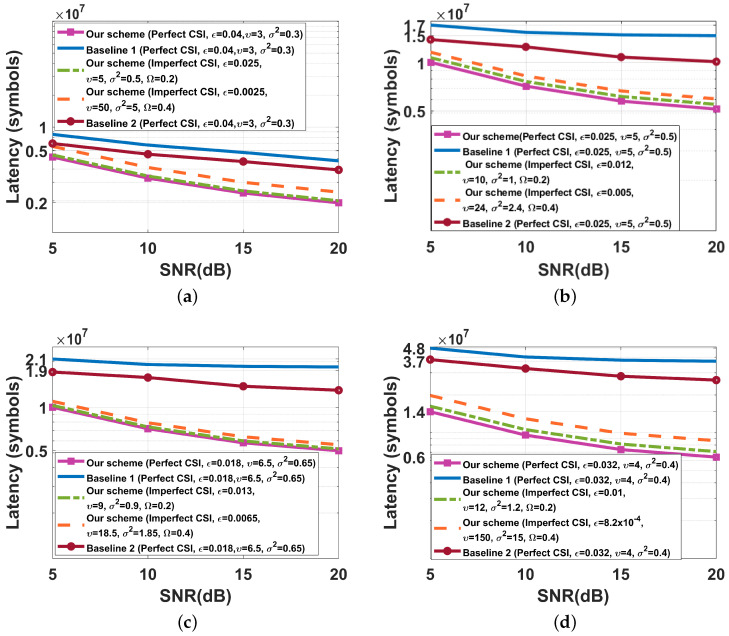
Transmission latency (200 rounds) of the different schemes on the MNIST dataset (K=10, $S_{\ell }=60,000$, q=15,910, $D=10^{-4}$, $\tau =10^{-6}$, $\tilde{\mathrm{SNR}}=15$ dB, $\sigma_{1}^{2}=1,\;\sigma_{2}^{2}=1,\;$$\sigma_{e}^{2}=2$, T=200). (**a**) A=B=C=4, δ=0.99994. (**b**) A=1, B=C=4, δ=0.99997. (**c**) A=4, B=C=1, δ=0.99997. (**d**) A=B=C=1, δ=0.99998.

As depicted in Figure 9, the transmission latency of our FBL scheme is approximately 2 to 5 times lower than that of Baseline 1 and Baseline 2, due to the gain from introducing feedback. Additionally, the transmission latency of our scheme decreases as the number of antennas increases. Furthermore, the transmission latency of Baseline 2 is lower than that of Baseline 1, owing to the gain from introducing AN to counter eavesdropping attacks in Baseline 2. Moreover, Figure 9 shows that the transmission latency of our FBL scheme increases as the maximum normalized estimation error of the eavesdropper’s channel increases, and this is because to support the same level of performance, the worse estimation of the CSI of the eavesdropper’s channel, the more bits need to be transmitted, which leads to an increase in transmission latency.

**Figure 10:**
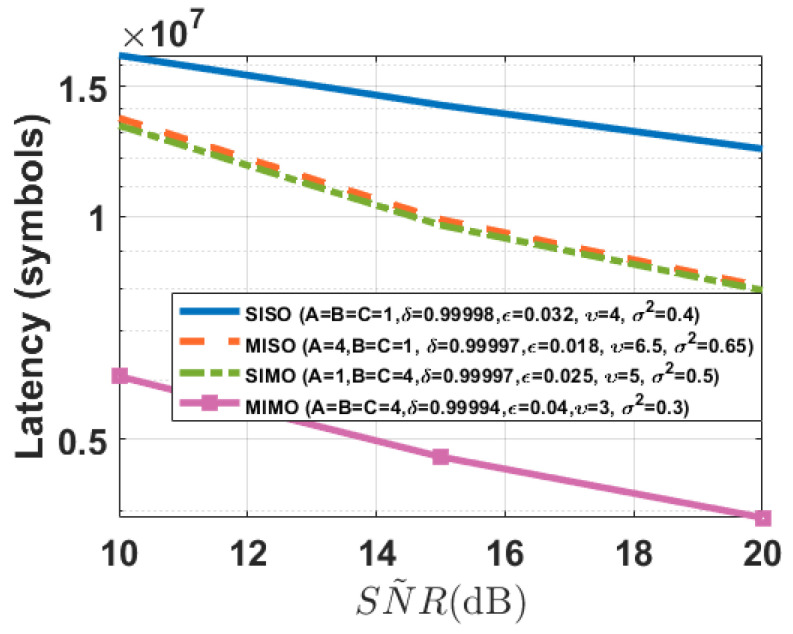
Transmission latency (200 rounds) of our schemes under different feedback channel SNR and perfect CSI on the MNIST dataset (K=10, $S_{\ell }=60,000$, q=15,910, P=10, $D=10^{-4}$, $\tau =10^{-6}$, $\sigma_{1}^{2}=1,\;\sigma_{2}^{2}=1,\;\sigma_{e}^{2}=2$, T=200).

From Table 2, we show that the achievable secrecy transmission rates of our FBL schemes increase with the number of antennas, and the achievable secrecy transmission rates of our schemes are significantly higher than those of Baseline 1 and Baseline 2, due to the gain introduced by feedback in our scheme. Additionally, due to the gain from introducing AN in Baseline 2, its achievable secrecy transmission rate is higher than that of Baseline 1. Furthermore, Table 2 shows that the achievable secrecy transmission rates of our proposed FBL scheme decrease as the maximum normalized estimation errors of the eavesdropper’s channel increase, which can be viewed as the price for the worst estimation. From Table 3, we conclude that the achievable secrecy transmission rates of FBL schemes increase as the SNR of the feedback channel increases. Moreover, Figure 10 shows that the transmission latency of FBL schemes increases as the SNR of the feedback channel decreases. Therefore, in our schemes, poorer feedback channel conditions lead to lower achievable secrecy transmission rates and increased transmission latency. However, poorer feedback channel conditions do not directly affect learning performance, as it is primarily determined by the distortion *D* of lossy source coding, the average decoding error probability τ of channel coding, and the variance of noise introduced by LDP mechanisms.

**Figure 11:**
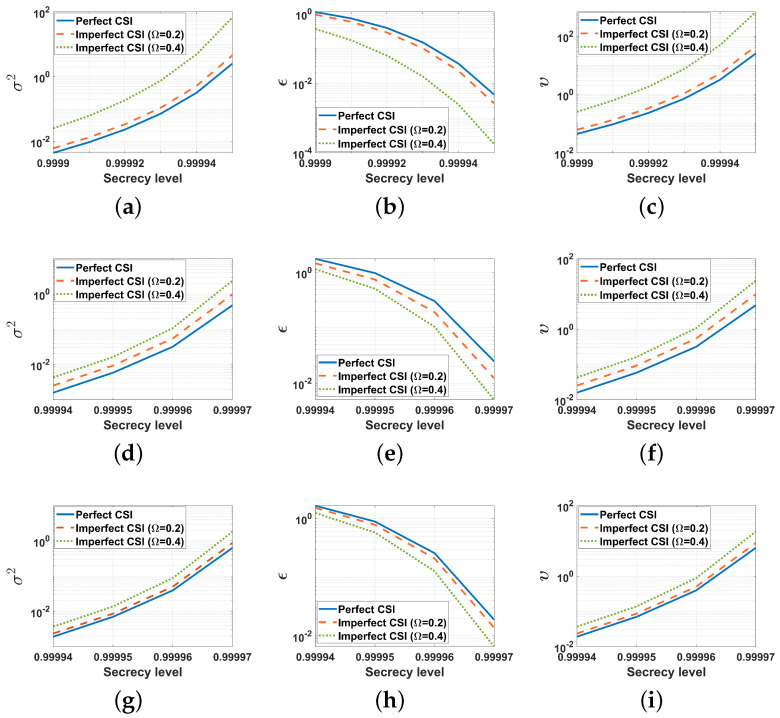

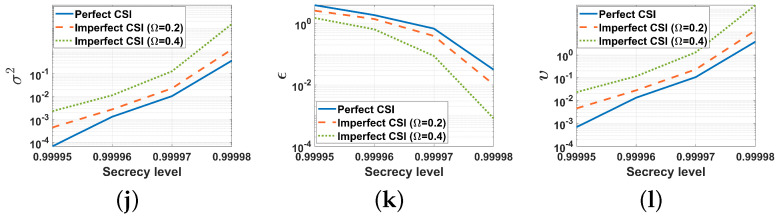
The relationship between the PLS (secrecy level), the privacy-utility, and LDP noise variance of proposed FBL schemes on the MNIST dataset (K=10, $S_{\ell }=60,000$, q=15,910, $D=10^{-4}$, $\tilde{\mathrm{SNR}}=15$ dB, P=10, $\tau =10^{-6}$, $\sigma_{1}^{2}=\sigma_{2}^{2}=1,\;$$\sigma_{e}^{2}=2$). (**a**) A=B=C=4. (**b**) A=B=C=4. (**c**) A=B=C=4. (**d**) A=1, B=C=4. (**e**) A=1, B=C=4. (**f**) A=1, B=C=4. (**g**) A=4, B=C=1. (**h**) A=4, B=C=1. (**i**) A=4, B=C=1. (**j**) A=B=C=1. (**k**) A=B=C=1. (**l**) A=B=C=1.

Figure 11 shows the relationship between PLS (measured by the secrecy level), privacy, utility, and the LDP noise variance of proposed FBL schemes. From Figure 11, we conclude that the secrecy level increases as the LDP noise variance increases, and a higher secrecy level leads to a more stringent relationship between privacy and utility (with a smaller ϵ and a larger υ). Apart from this, for a given secrecy level, increasing the maximum normalized estimation error in the eavesdropper’s channel results in an increase in the variance of LDP noise, which can be also viewed as the price for the worse estimation.

## 6. Conclusions and Future Work

In this paper, a practical FBL approach, which is an extension of the classical SK scheme, is proposed for the multi-antenna URLLC-WHFL systems in the presence of PLS. We characterize the relationship between PLS, privacy, and the utility of these WHFL systems, and derive achievable transmission rates of the proposed FBL approach. Simulation results demonstrate that when the edge server has perfect knowledge of the eavesdropper’s CSI, our proposed FBL approach not only almost achieves perfect secrecy but also does not affect learning performance. Additionally, simulation results demonstrate that the proposed schemes have robustness even when the edge server has an imperfect eavesdropper’s CSI. Apart from this, it has been demonstrated that the transmission latency of our proposed FBL approach is significantly lower compared to traditional RBCS.

Furthermore, this paper focuses on proposing and analyzing a theoretical scheme. The application of this approach in real-world systems still faces practical challenges, such as hardware constraints, power consumption, or synchronization issues. Future work should aim to optimize energy efficiency and address synchronization in more complex multi-antenna systems using the proposed FBL scheme. On the other hand, as the computational complexity of techniques like precoding, beamforming, and SVD increases with the number of devices and communication channels, particularly in multi-antenna systems, further research, and optimization are needed to extend our proposed approach to more complex and large-scale networks. For instance, distributed or hierarchical architectures can allocate the computational load across multiple servers or devices, reducing the burden on individual components. Additionally, low-complexity approximation methods for precoding and beamforming could help lower overall system complexity. Future work will extend our approach to more complex multi-edge scenarios, exploring the impact of interference among edge servers on the WHFL in the presence of PLS.

## Figures and Tables

**Table 1:** Summarizing all results in WFL in the presence of privacy, utility, PLS and URLLC.

Related Work	Privacy	Utility	PLS	Relationship between PLS, Privacy, and Utility	URLLC
[7,8,9,16,23]	✓	−	−	−	−
[10,11,12,13,14,22]	✓	✓	−	Relationship between Privacy and Utility	−
[26,27,28]	−	−	✓	−	−
[29]	−	✓	✓	Relationship between PLS and Utility	−
[30]	✓	✓	✓	Relationship between PLS-Privacy-Utility	−
[33,34]	−	−	−	−	✓
This Work	✓	✓	✓	Relationship between PLS-Privacy-Utility	✓

**Table 2:** Achievable secrecy rates of the different schemes on the MNIST dataset (K=10, $S_{\ell }=60,000$, q=15,910, $\tilde{\mathrm{SNR}}=15$ dB, P=10, $\tau =10^{-6}$, T=200, $D=10^{-4}$, $\sigma_{1}^{2}=\sigma_{2}^{2}=1,\;\sigma_{e}^{2}=2$).

Number of Antennas	A=B=C=4 (MIMO)	A=1,B=C=4 (SIMO)	A=4,B=C=1 (MISO)	A=B=C=1 (SISO)
Our scheme (Perfect CSI)	10.3951(bits/symbol) (ϵ=0.04, υ=3, $\sigma^{2}=0.3$, δ=0.99994)	4.9928(bits/symbol) (ϵ=0.025, υ=5, $\sigma^{2}=0.5$, δ=0.99997)	5.1346(bits/symbol) (ϵ=0.018, υ=6.5, $\sigma^{2}=0.65$, δ=0.99997)	2.6718 (bits/symbol) (ϵ=0.032, υ=4, $\sigma^{2}=0.4$, δ=0.99998)
Our scheme (Imperfect CSI, Ω=0.2)	10.3941(bits/symbol) (ϵ=0.025, υ=5, $\sigma^{2}=0.5$, δ=0.99994)	4.9922(bits/symbol) (ϵ=0.012, υ=10, $\sigma^{2}=1$, δ=0.99997)	5.1343(bits/symbol) (ϵ=0.013, υ=9, $\sigma^{2}=0.9$, δ=0.99997)	2.6708 (bits/symbol) (ϵ=0.01, υ=12, $\sigma^{2}=1.2$, δ=0.99998)
Our scheme (Imperfect CSI, Ω=0.4)	10.3898(bits/symbol) (ϵ=0.0025, υ=50, $\sigma^{2}=5$, δ=0.99994)	4.9914(bits/symbol) (ϵ=0.005, υ=24, $\sigma^{2}=2.4$, δ=0.99997)	5.1338(bits/symbol) (ϵ=0.0065, υ=18.5, $\sigma^{2}=1.85$, δ=0.99997)	2.6689 (bits/symbol) ($\epsilon =8.2\times 10^{-4}$, υ=150, $\sigma^{2}=15$, δ=0.99998)
Baseline 1 [26,28] (Perfect CSI)	4.2827(bits/symbol) (ϵ=0.04, υ=3, $\sigma^{2}=0.3$)	2.1537(bits/symbol) (ϵ=0.025, υ=5, $\sigma^{2}=0.5$)	2.2124(bits/symbol) (ϵ=0.018, υ=6.5, $\sigma^{2}=0.65$)	0.8046(bits/symbol) (ϵ=0.032, υ=4, $\sigma^{2}=0.4$)
Baseline 2 [29] (Perfect CSI)	5.2228(bits/symbol) (ϵ=0.04, υ=3, $\sigma^{2}=0.3$)	2.6265(bits/symbol) (ϵ=0.025, υ=5, $\sigma^{2}=0.5$)	2.6981(bits/symbol) (ϵ=0.018, υ=6.5, $\sigma^{2}=0.65$)	0.9812(bits/symbol) (ϵ=0.032, υ=4, $\sigma^{2}=0.4$)

**Table 3:** Achievable secrecy rates of our schemes under different feedback channel SNR on the MNIST dataset (K=10, $S_{\ell }=60,000$, q=15,910, P=10, $\tau =10^{-6}$, T=200, $D=10^{-4}$, $\sigma_{1}^{2}=\sigma_{2}^{2}=1,\;\sigma_{e}^{2}=2$).

Number of Antennas	A=B=C=4 (MIMO) (ϵ=0.04, υ=3, $\sigma^{2}=0.3$, δ=0.99994)	A=1,B=C=4 (SIMO) (ϵ=0.025, υ=5, $\sigma^{2}=0.5$, δ=0.99997)	A=4,B=C=1 (MISO) (ϵ=0.018, υ=6.5, $\sigma^{2}=0.65$, δ=0.99997)	A=B=C=1 (SISO) (ϵ=0.032, υ=4, $\sigma^{2}=0.4$, δ=0.99998)
$\tilde{\mathrm{SNR}}=10$ dB (Perfect CSI)	8.0911(bits/symbol)	3.6612(bits/symbol)	3.7589(bits/symbol)	2.2885(bits/symbol)
$\tilde{\mathrm{SNR}}=15$ dB (Perfect CSI)	10.3951(bits/symbol)	4.9928(bits/symbol)	5.1346(bits/symbol)	2.6718(bits/symbol)
$\tilde{\mathrm{SNR}}=20$ dB (Perfect CSI)	12.5581(bits/symbol)	6.1146(bits/symbol)	6.3177(bits/symbol)	3.0622(bits/symbol)

## Data Availability

The raw data supporting the conclusions of this article will be made available by the authors on request.

## Appendix A. The Formal Proof of Theorem 1

### Appendix A.1. Utility and Privacy Analysis

First, note that since $W_{t}$, $\eta_{t}$, and $W_{t}^{\prime }$ are i.i.d. generated, from Definition 1, we conclude the following:

(A1) $$\begin{array}{c} \max_{t\in \{1,\ldots ,T\}}\frac{1}{q}I\left(W_{t};W_{t}^{\prime }\right)=\max_{t\in \{1,\ldots ,T\}}\frac{1}{2}\log\left(1+\frac{S_{\ell }\sigma_{w,t}^{2}}{K\sigma^{2}}\right)\leq \epsilon , \end{array}$$

On the other hand, from Definition 2, we conclude the following:

(A2) $$\begin{array}{c} \frac{1}{qT}\sum_{t=1}^{T}E\left(d\left(W_{t},W_{t}^{\prime }\right)\right)=\frac{1}{qT}\sum_{t=1}^{T}E(||\eta_{t}{\left|\right|}^{2})=K\sigma^{2}\leq \upsilon . \end{array}$$

From (A1) and (A2), the relationship between privacy, utility, and the noise variance of LDP is characterized by the following:

(A3) $$\underset{\mathrm{Privacy}\mathrm{term}}{\underset{︸}{\max_{t\in \{1,\ldots ,T\}}\frac{S_{\ell }\sigma_{w,t}^{2}}{K(2^{2\epsilon }-1)}}}\leq \underset{\begin{array}{c} \mathrm{Noise}\mathrm{variance}\mathrm{of}\mathrm{LDP} \end{array}}{\underset{︸}{\sigma^{2}}}\leq \underset{\begin{array}{c} \mathrm{Utility}\mathrm{term} \end{array}}{\underset{︸}{\frac{\upsilon }{K}}},$$

which indicates that by selecting an appropriate LDP noise to satisfy (A3), both privacy and utility can be ensured.

### Appendix A.2. Decoding Error Probability and Convergence Analysis

First, we bound the decoding error probability $P_{e,t}$ of $W_{t}^{\prime \prime }$ transmitted in all parallel sub-SISO channels as follows:

(A4) $$\begin{array}{c} P_{e,t}\leq P_{e,t}\left(1\right)+P_{e,t}\left(2\right)+\ldots +P_{e,t}\left(J\right), \end{array}$$

where $P_{e,t}\left(j\right)$ (j=1,…,J) represents the decoding error probability of message $W_{t,j}^{\prime \prime }$, and *J* is the number of parallel sub-SISO channels, which is defined in (31). Next, we analyze the error events of message $W_{t,j}^{\prime \prime }$, which consist of the following:

(1) A modulo-aliasing error occurs in the edge server at time instant $i+1(1\leq i\leq N_{t}-1)$, and it is defined as follows:
  (A5) $$\begin{array}{c} E_{i}=\left\{\gamma_{i}\varepsilon_{i}+\frac{\eta_{j,2,i}^{\prime }\left(t\right)}{{\tilde{d}}_{j}}\notin \Lambda \right\}=\left\{\left[\gamma_{i}\varepsilon_{R,i}+\frac{\eta_{R,j,2,i}^{\prime }\left(t\right)}{{\tilde{d}}_{j}}\notin \left[-\frac{d}{2},\frac{d}{2}\right)\right]\cup \left[\gamma_{i}\varepsilon_{I,i}+\frac{\eta_{I,j,2,i}^{\prime }\left(t\right)}{{\tilde{d}}_{j}}\notin \left[-\frac{d}{2},\frac{d}{2}\right)\right]\right\}, \end{array}$$
  where $\varepsilon_{R,i}$ and $\varepsilon_{I,i}$ are the real and imaginary parts of $\varepsilon_{i}$, respectively, $\eta_{R,j,2,i}^{\prime }\left(t\right)$ and $\eta_{I,j,2,i}^{\prime }\left(t\right)$ are the real and imaginary parts of $\eta_{j,2,i}^{\prime }\left(t\right)$, respectively.
(2) A decoding error occurs in the cloud server at time instant $N_{t}$, and it is defined as follows:
  (A6) $$\begin{array}{c} E_{N_{t}}=\left\{\varepsilon_{R,N_{t}}\notin \left[-\frac{\sqrt{3}}{2^{N_{t}R_{t,j,R}}},\frac{\sqrt{3}}{2^{N_{t}R_{t,j,R}}}\right)\cup \varepsilon_{I,N_{t}}\notin \left[-\frac{\sqrt{3}}{2^{N_{t}R_{t,j,I}}},\frac{\sqrt{3}}{2^{N_{t}R_{t,j,I}}}\right)\right\}, \end{array}$$
  where $\varepsilon_{R,N_{t}}$ and $\varepsilon_{I,N_{t}}$ are the real and imaginary parts of $\varepsilon_{N_{t}}$, respectively.

Thus, the error probability $P_{e,t}\left(j\right)$ is bounded by the following:

(A7) $$\begin{array}{cc} P_{e,t}\left(j\right) & \leq Pr\left(\overset{N_{t}}{\underset{i=1}{⋃}}E_{i}\right)=Pr\left(\overset{N_{t}-1}{\underset{i=1}{⋃}}E_{i}\right)+Pr\left(\overset{N_{t}-1}{\underset{i=1}{⋂}}E_{i}^{c}\cap E_{N_{t}}\right)\\ \; & =\sum_{i=1}^{N_{t}-1}Pr\left(\overset{i-1}{\underset{j=1}{⋂}}E_{j}^{c}\cap E_{i}\right)+Pr\left(\overset{N_{t}-1}{\underset{i=1}{⋂}}E_{i}^{c}\cap E_{N_{t}}\right)\\ \; & =\sum_{i=1}^{N_{t}-1}Pr\left({\tilde{E}}_{i}\right)+Pr\left({\tilde{E}}_{N_{t}}\right), \end{array}$$

where $E^{c}$ is the complement of the set *E*, and ${\tilde{E}}_{i}={⋂}_{j=1}^{i-1}E_{j}^{c}\cap E_{i}$. Here, note that $Pr\left({\tilde{E}}_{i}\right)\left(i\in \left\{1,\ldots ,N_{t}-1\right\}\right)$ is the error probability that a demodulation error occurs at time instant i+1, and no error occurs in all previous times. $Pr({\tilde{E}}_{N_{t}})$ is the error probability of the final decoding, and no demodulation error occurs in all times. We assume that $P_{e,t}\leq \tau$, which indicates that $\frac{1}{T}\sum_{t=1}^{T}P_{e,t}\leq \tau$ is guaranteed. Then, we choose $P_{e,t}\left(j\right)\leq \frac{\tau }{J}$, and we have the following:

(A8) $$\begin{array}{c} Pr\left({\tilde{E}}_{N_{t}}\right)=\sum_{i=1}^{N_{t}-1}Pr\left({\tilde{E}}_{i}\right)=\frac{\tau }{2J}, \end{array}$$

for simplification, we define the following:

(A9) $$\begin{array}{c} Pr\left({\tilde{E}}_{1}\right)=\ldots =Pr\left({\tilde{E}}_{N_{t}-1}\right)=p_{m}. \end{array}$$

Substituting (A9) into (A8), we have the following:

(A10) $$\begin{array}{c} p_{m}=\frac{\tau }{2J(N_{t}-1)}. \end{array}$$

For the error event ${\tilde{E}}_{i}$, since no demodulation error occurs before time instant i+1, according to (43), (44), and the fact $\eta_{j,2,i}^{\prime }\left(t\right)$ is CSCG-distributed, we can conclude the following: $\gamma_{i}\varepsilon_{i}+\frac{\eta_{j,2,i}^{\prime }\left(t\right)}{{\tilde{d}}_{j}}∼\mathrm{CN}\left(0,\gamma_{i}^{2}\alpha_{i}+\frac{\sigma_{2}^{2}}{{\tilde{d}}_{j}^{2}}\right)$. Hence, we have $\gamma_{i}\varepsilon_{k,i}+\frac{\eta_{k,j,2,i}^{\prime }\left(t\right)}{{\tilde{d}}_{j}}∼N\left(0,\gamma_{i}^{2}\frac{\alpha_{i}}{2}+\frac{\sigma_{2}^{2}}{2{\tilde{d}}_{j}^{2}}\right)$, where k∈{R,I}. From (A5) and $d=\sqrt{6{\tilde{P}}_{j}}$, we have the following:

(A11) $$\begin{array}{cc} Pr(E_{i}) & =Pr(\left\{\left[\gamma_{i}\varepsilon_{R,i}+\frac{\eta_{R,j,2,i}^{\prime }\left(t\right)}{{\tilde{d}}_{j}}\notin \left[-\frac{d}{2},\frac{d}{2}\right)\right]\cup \left[\gamma_{i}\varepsilon_{I,i}+\frac{\eta_{I,j,2,i}^{\prime }\left(t\right)}{{\tilde{d}}_{j}}\notin \left[-\frac{d}{2},\frac{d}{2}\right)\right]\right\})\\ \; & \leq Pr\left(\gamma_{i}\varepsilon_{R,i}+\frac{\eta_{R,j,2,i}^{\prime }\left(t\right)}{{\tilde{d}}_{j}}\notin \left[-\frac{d}{2},\frac{d}{2}\right)\right)+Pr\left(\gamma_{i}\varepsilon_{I,i}+\frac{\eta_{I,j,2,i}^{\prime }\left(t\right)}{{\tilde{d}}_{j}}\notin \left[-\frac{d}{2},\frac{d}{2}\right)\right)\\ \; & =2Q\left(\sqrt{\frac{\frac{3{\tilde{P}}_{j}}{2}}{E{\left(\gamma_{i}\varepsilon_{R,i}+\frac{\eta_{R,j,2,i}^{\prime }\left(t\right)}{{\tilde{d}}_{j}}\right)}^{2}}}\right)+2Q\left(\sqrt{\frac{\frac{3{\tilde{P}}_{j}}{2}}{E{\left(\gamma_{i}\varepsilon_{I,i}+\frac{\eta_{I,j,2,i}^{\prime }\left(t\right)}{{\tilde{d}}_{j}}\right)}^{2}}}\right)\\ \; & =4Q\left(\sqrt{\frac{\frac{3{\tilde{P}}_{j}}{2}}{\gamma_{i}^{2}\frac{\alpha_{i}}{2}+\frac{\sigma_{2}^{2}}{2{\tilde{d}}_{j}^{2}}}}\right)=p_{m}. \end{array}$$

For simplification, let

(A12) $$\begin{array}{c} \xi =\frac{1}{3}{\left[Q^{-1}\left(\frac{p_{m}}{4}\right)\right]}^{2}=\frac{1}{3}{\left[Q^{-1}\left(\frac{\tau }{8J(N_{t}-1)}\right)\right]}^{2}. \end{array}$$

Substituting (A11) into (A12), we have the following:

(A13) $$\begin{array}{c} \gamma_{i}^{2}\frac{\alpha_{i}}{2}+\frac{\sigma_{2}^{2}}{2{\tilde{d}}_{j}^{2}}=\frac{{\tilde{P}}_{j}}{2\xi }. \end{array}$$

From (A13), we can conclude the following:

(A14) $$\begin{array}{c} \gamma_{i}=\sqrt{\frac{1}{\alpha_{i}}\left(\frac{{\tilde{P}}_{j}}{\xi }-\frac{\sigma_{2}^{2}}{{\tilde{d}}_{j}^{2}}\right)}, \end{array}$$

and note that $X_{j,i+1}^{\prime }\left(t\right)$ is subject to the power constraint $P_{j}$; hence, we have the following:

(A15) $$\begin{array}{c} E\left[X_{j,i+1}^{\prime }{\left(t\right)}^{H}X_{j,i+1}^{\prime }\left(t\right)\right]=\lambda_{i}^{2}E{\left(\gamma_{i}\varepsilon_{i}+\frac{\eta_{j,2,i}^{\prime }\left(t\right)}{{\tilde{d}}_{j}}\right)}^{2}=P_{j}. \end{array}$$

According to (A13), (A15), we conclude the following:

(A16) $$\begin{array}{c} \lambda_{i}=\sqrt{\xi \cdot \frac{P_{j}}{{\tilde{P}}_{j}}}. \end{array}$$

From (43), (A14), and (A16), we have the following:

(A17) $$\begin{array}{c} Y_{j,i+1}^{\prime }\left(t\right)=d_{j}\lambda_{i}\left(\gamma_{i}\varepsilon_{i}+\frac{\eta_{j,2,i}^{\prime }\left(t\right)}{{\tilde{d}}_{j}}\right)+\eta_{j,1,i+1}^{\prime }\left(t\right), \end{array}$$

we have the following:

(A18) $$\begin{array}{cc} \beta_{i+1} & =\frac{\lambda_{i}\gamma_{i}\alpha_{i}}{P_{j}+\frac{\sigma_{1}^{2}}{d_{j}^{2}}}=\frac{\sqrt{\alpha_{i}}}{\sigma_{1}}\frac{\sqrt{{\mathrm{SNR}}_{j}\left(1-\xi \cdot {\tilde{\mathrm{SNR}}}_{j}^{-1}{\tilde{d}}_{j}^{-2}\right)}}{{\mathrm{SNR}}_{j}+d_{j}^{-2}}. \end{array}$$

According to (44), (A14), (A16), (A17), and (A18), we have the following:

(A19) $$\begin{array}{cc} \alpha_{i+1} & =\alpha_{i}{\left(1+{\mathrm{SNR}}_{j}d_{j}^{2}\frac{1-\xi \cdot {\tilde{\mathrm{SNR}}}_{j}^{-1}{\tilde{d}}_{j}^{-2}}{1+\xi \cdot {\mathrm{SNR}}_{j}\cdot {\tilde{\mathrm{SNR}}}_{j}^{-1}d_{j}^{2}{\tilde{d}}_{j}^{-2}}\right)}^{-1}=2d_{j}^{-2}{\mathrm{SNR}}_{j}^{-1}{\left(1+\frac{{\mathrm{SNR}}_{j}d_{j}^{2}}{\Psi_{1}\Psi_{2}}\right)}^{-i}, \end{array}$$

where $\Psi_{1}$, $\Psi_{2}$, and ξ are defined in (19). From (A6)–(A8), we have the following:

(A20) $$\begin{array}{cc} Pr\left({\tilde{E}}_{N_{t}}\right) & \leq Pr\left\{\varepsilon_{R,N_{t}}\notin \left[-\frac{\sqrt{3}}{2^{N_{t}R_{t,j,R}}},\frac{\sqrt{3}}{2^{N_{t}R_{t,j,R}}}\right)\right\}+Pr\left\{\varepsilon_{I,N_{t}}\notin \left[-\frac{\sqrt{3}}{2^{N_{t}R_{t,j,I}}},\frac{\sqrt{3}}{2^{N_{t}R_{t,j,I}}}\right)\right\}\leq \frac{\tau }{2J}, \end{array}$$

and let

(A21) $$\begin{array}{c} Pr\left\{\varepsilon_{R,N_{t}}\notin \left[-\frac{\sqrt{3}}{2^{N_{t}R_{t,j,R}}},\frac{\sqrt{3}}{2^{N_{t}R_{t,j,R}}}\right)\right\}=Pr\left\{\varepsilon_{I,N_{t}}\notin \left[-\frac{\sqrt{3}}{2^{N_{t}R_{t,j,I}}},\frac{\sqrt{3}}{2^{N_{t}R_{t,j,I}}}\right)\right\}=\frac{\tau }{4J}. \end{array}$$

Next, we first analyze the term $Pr\left\{\varepsilon_{R,N_{t}}\notin \left[-\frac{\sqrt{3}}{2^{N_{t}R_{t,j,R}}},\frac{\sqrt{3}}{2^{N_{t}R_{t,j,R}}}\right)\right\}=\frac{\tau }{4J}$, i.e.,

(A22) $$\begin{array}{c} Pr\left\{\varepsilon_{R,N_{t}}\notin \left[-\frac{\sqrt{3}}{2^{N_{t}R_{t,j,R}}},\frac{\sqrt{3}}{2^{N_{t}R_{t,j,R}}}\right)\right\}=2Q\left(\frac{\sqrt{3}}{2^{N_{t}R_{t,j,R}}}\cdot \frac{1}{\sqrt{\alpha_{R,N_{t}}}}\right)=2Q\left(\frac{\sqrt{3}}{2^{N_{t}R_{t,j,R}}}\cdot \frac{1}{\sqrt{\frac{\alpha_{N_{t}}}{2}}}\right)=\frac{\tau }{4J}. \end{array}$$

Substituting (A19) into (A22), we have the following:

(A23) $$\begin{array}{c} R_{t,j,R}=\frac{1}{2N_{t}}\log\left(\frac{3{\mathrm{SNR}}_{j}d_{j}^{2}}{{\left[Q^{-1}\left(\frac{\tau }{8J}\right)\right]}^{2}}{\left(1+\frac{{\mathrm{SNR}}_{j}d_{j}^{2}}{\Psi_{1}\Psi_{2}}\right)}^{N_{t}-1}\right). \end{array}$$

Analogously, we can show that $R_{t,j,I}=R_{t,j,R}$. The transmission rate of the *j*-th (j=1,…,J) parallel sub-channel in the *t*-th round is given by the following:

(A24) $$R_{t,j}=R_{t,j,R}+R_{t,j,I}=\frac{1}{N_{t}}\log\left(\frac{3{\mathrm{SNR}}_{j}d_{j}^{2}}{{\left[Q^{-1}\left(\frac{\tau }{8J}\right)\right]}^{2}}{\left(1+\frac{{\mathrm{SNR}}_{j}d_{j}^{2}}{\Psi_{1}\Psi_{2}}\right)}^{N_{t}-1}\right).$$

Combining (A24) and (36), and power allocating in Section 3.1.1, (18) in Theorem 1 is obtained. Then, according to $R=\frac{H(W_{1}^{\prime \prime },\ldots ,W_{T}^{\prime \prime })}{N}$ in (15), the transmission rate $R_{mimo}\left(\tau ,N,\delta ,D,\upsilon ,\epsilon \right)$ is given by the following:

(A25) $$\begin{array}{c} R_{mimo}\left(\tau ,N,\delta ,D,\upsilon ,\epsilon \right)=\frac{H(W_{1}^{\prime \prime },\ldots ,W_{T}^{\prime \prime })}{N}\overset{\left(b\right)}{=}\frac{\sum_{t=1}^{T}H\left(W_{t}^{\prime \prime }\right)}{N}=\frac{\sum_{t=1}^{T}N_{t}R_{t}}{N}, \end{array}$$

where $N=\sum_{t=1}^{T}N_{t}$, and (b) is due to the fact that $W_{t}^{\prime }$ is mapped into the uniformly distributed index $W_{t}^{\prime \prime }$ in each communication round, which indicates that $\left(W_{1}^{\prime \prime },\ldots ,W_{T}^{\prime \prime }\right)$ are independent of each other; hence, (17) in Theorem 1 is obtained. A brief convergence analysis of our FBL approach is given below.

*Convergence analysis*: Following the convergence proof in [18,22], we can prove the existence of convergence in the WHFL system with our FBL scheme when the decoding error probability of our FBL scheme is significantly small. Furthermore, from (A19) and (A22), we conclude that the variance of the estimation error in our FBL approach *converges to zero with double-exponential speed*, which indicates that the final estimation ${\hat{\theta }}_{j,N_{t}}$ can always converge to $\theta_{j}$, and the required coding block length for achieving the desired decoding error probability is significantly short.

### Appendix A.3. Security Analysis

First, note that the eavesdropper’s equivocation rate, Δ, can be re-written as follows:

(A26) $$\begin{array}{cc} \Delta & =\frac{H(W_{1}^{\prime \prime },\ldots ,W_{T}^{\prime \prime }|Z^{N_{1}},\ldots ,Z^{N_{T}},h_{mimo},{\tilde{h}}_{mimo},g_{mimo},{\tilde{g}}_{mimo})}{H(W_{1}^{\prime \prime },\ldots ,W_{T}^{\prime \prime })}\\ \; & =\frac{\sum_{t=1}^{T}H\left(W_{t}^{\prime \prime }|Z^{N_{1}},\ldots ,Z^{N_{T}},h_{mimo},{\tilde{h}}_{mimo},g_{mimo},{\tilde{g}}_{mimo},W_{1}^{\prime \prime },\ldots ,W_{t-1}^{\prime \prime }\right)}{H(W_{1}^{\prime \prime },\ldots ,W_{T}^{\prime \prime })}\\ \; & \overset{\left(c\right)}{=}\frac{\sum_{t=1}^{T}H\left(W_{t}^{\prime \prime }|Z^{N_{t}},h_{mimo},{\tilde{h}}_{mimo},g_{mimo},{\tilde{g}}_{mimo}\right)}{H(W_{1}^{\prime \prime },\ldots ,W_{T}^{\prime \prime })}=\frac{\sum_{t=1}^{T}H\left(W_{t}^{\prime \prime }|Z^{N_{t}},h_{mimo},{\tilde{h}}_{mimo},g_{mimo},{\tilde{g}}_{mimo}\right)}{\sum_{t=1}^{T}H\left(W_{t}^{\prime \prime }\right)}, \end{array}$$

where (c) follows from the Markov chain $W_{t}^{\prime \prime }\to \left(Z^{N_{t}},h_{mimo},{\tilde{h}}_{mimo},g_{mimo},{\tilde{g}}_{mimo}\right)\to (Z^{N_{1}},\ldots ,$ $Z^{N_{t-1}},Z^{N_{t+1}},\ldots ,Z^{N_{T}},W_{1}^{\prime \prime },\ldots ,W_{t-1}^{\prime \prime })$. The term $H(W_{t}^{\prime \prime }|Z^{N_{t}},h_{mimo},{\tilde{h}}_{mimo},g_{mimo},{\tilde{g}}_{mimo})$ in (A26) is given by the following:

(A27) $$\begin{array}{cc} \; & H(W_{t}^{\prime \prime }|Z^{N_{t}},h_{mimo},{\tilde{h}}_{mimo},g_{mimo},{\tilde{g}}_{mimo})\\ \; & \overset{\left(e\right)}{\geq }H\left(W_{t}^{\prime \prime }\right|\underset{Z_{1}\left(t\right)}{\underset{︸}{g_{mimo}X_{1}\left(t\right)+{\tilde{g}}_{mimo}{\tilde{X}}_{1}\left(t\right)+\eta_{e,1}\left(t\right)}},\ldots ,\underset{Z_{N_{t}-1}\left(t\right)}{\underset{︸}{g_{mimo}X_{N_{t}-1}\left(t\right)+{\tilde{g}}_{mimo}{\tilde{X}}_{N_{t}-1}\left(t\right)+\eta_{e,N_{t}-1}\left(t\right)}},\\ \; & \underset{Z_{N_{t}}\left(t\right)}{\underset{︸}{g_{mimo}X_{N_{t}}\left(t\right)+\eta_{e,N_{t}}\left(t\right)}},\eta_{1,1}\left(t\right),\ldots ,\eta_{1,N_{t}}\left(t\right),\eta_{2,1}\left(t\right),\ldots ,\eta_{2,N_{t}}\left(t\right),\eta_{e,2}\left(t\right),\ldots ,\eta_{e,N_{t}}\left(t\right),{\tilde{X}}_{1}^{\prime }\left(t\right),\ldots ,{\tilde{X}}_{N_{t}-1}^{\prime }\left(t\right),h_{mimo},\\ \; & {\tilde{h}}_{mimo},g_{mimo},{\tilde{g}}_{mimo},U,\Lambda ,V,\tilde{U},\tilde{\Lambda },\tilde{V})\\ \; & \overset{\left(f\right)}{=}H\left(W_{t}^{\prime \prime }\right|g_{mimo}VX_{1}^{\prime }\left(t\right)+\eta_{e,1}\left(t\right),\eta_{1,1}\left(t\right),\ldots ,\eta_{1,N_{t}}\left(t\right),\eta_{2,1}\left(t\right),\ldots ,\eta_{2,N_{t}}\left(t\right),\eta_{e,2}\left(t\right),\ldots ,\eta_{e,N_{t}}\left(t\right),{\tilde{X}}_{1}^{\prime }\left(t\right),\ldots ,{\tilde{X}}_{N_{t}-1}^{\prime }\left(t\right),\\ \; & h_{mimo},{\tilde{h}}_{mimo},g_{mimo},{\tilde{g}}_{mimo},U,\Lambda ,V,\tilde{U},\tilde{\Lambda },\tilde{V})\\ \; & \overset{\left(g\right)}{=}H\left(W_{t}^{\prime \prime }|g_{mimo}VX_{1}^{\prime }\left(t\right)+\eta_{e,1}\left(t\right)\right)\overset{\left(h\right)}{=}H\left(W_{t}^{\prime \prime }\right)+h\left(\eta_{e,1}\left(t\right)\right)-h\left(g_{mimo}VX_{1}^{\prime }\left(t\right)+\eta_{e,1}\left(t\right)\right)\\ \; & \overset{\left(i\right)}{=}H\left(W_{t}^{\prime \prime }\right)-\underset{\mathrm{Information}\mathrm{leakage}\mathrm{at}\mathrm{time}1}{\underset{︸}{\log\det\left(I+\frac{g_{mimo}K_{x_{1}}g_{mimo}^{H}}{\sigma_{e}^{2}}\right)}}, \end{array}$$

where

(e) follows from the fact that conditioning reduces entropy, as shown in (28),

(f) follows from $X_{i}^{\prime }\left(t\right)=V^{H}X_{i}\left(t\right),{\tilde{X}}_{i}^{\prime }\left(t\right)={\tilde{V}}^{H}{\tilde{X}}_{i}\left(t\right)$ and $X_{i}^{\prime }\left(t\right)$$\left(i=2,\ldots ,N_{t}\right)$ is a function of $h_{mimo},{\tilde{h}}_{mimo},\eta_{1,1}\left(t\right),$$\ldots ,\eta_{1,i-1}\left(t\right),\eta_{2,1}\left(t\right),\ldots ,\eta_{2,i-1}\left(t\right)$,

(g) follows from the fact that ${\tilde{X}}_{i}^{\prime }\left(t\right)$$\left(i=1,\ldots ,N_{t}-1\right)$ is only related to $\nu_{i}$ [39] [Chapter 4.1, pp. 61–63], and $h_{mimo}$, ${\tilde{h}}_{mimo}$, $g_{mimo}$, ${\tilde{g}}_{mimo}$, U, Λ, V, $\tilde{U}$, $\tilde{\Lambda }$, $\tilde{V}$, $\eta_{1,1}\left(t\right),\ldots ,\eta_{1,N_{t}}\left(t\right)$, $\eta_{2,1}\left(t\right),\ldots ,\eta_{2,N_{t}}\left(t\right)$, $\eta_{e,2}\left(t\right),\ldots ,\eta_{e,N_{t}}\left(t\right)$, $\nu_{1},\ldots ,\nu_{N_{t}-1}$ are independent of $W_{t}^{\prime \prime }$, $X_{1}^{\prime }\left(t\right)$, $\eta_{e,1}\left(t\right)$,

(h) is due to the fact that (33) and $X_{j,1}^{\prime }\left(t\right)=\sqrt{\frac{P_{j}}{2}}\theta_{j}$, and $W_{t}^{\prime \prime }=(W_{t,1}^{\prime \prime },$$\ldots ,W_{t,J}^{\prime \prime })$ are mapped into $\left(\theta_{1},\ldots ,\theta_{J}\right)$, respectively,

(i) follows from the following:

(A28) $$\begin{array}{cc} h\left(g_{mimo}VX_{1}^{\prime }\left(t\right)+\eta_{e,1}\left(t\right)\right)-h\left(\eta_{e,1}\left(t\right)\right) & \leq \log\det\left(I+\frac{g_{mimo}VE\left(X_{1}^{\prime }\left(t\right)X_{1}^{\prime H}\left(t\right)\right)V^{H}g_{mimo}^{H}}{\sigma_{e}^{2}}\right)\\ \; & =\log\det\left(I+\frac{g_{mimo}E\left(X_{1}\left(t\right)X_{1}^{H}\left(t\right)\right)g_{mimo}^{H}}{\sigma_{e}^{2}}\right), \end{array}$$

and $K_{x_{1}}=E\left(X_{1}\left(t\right)X_{1}^{H}\left(t\right)\right)$. Substituting (A27) into (A26), we have the following:

(A29) $$\begin{array}{cc} \Delta & \geq \frac{\sum_{t=1}^{T}H\left(W_{t}^{\prime \prime }\right)\left(1-\frac{\log\det\left(I+\frac{g_{mimo}K_{x_{1}}g_{mimo}^{H}}{\sigma_{e}^{2}}\right)}{H(W_{t}^{\prime \prime })}\right)}{\sum_{t=1}^{T}H\left(W_{t}^{\prime \prime }\right)}\geq \min_{t\in \{1,\ldots ,T\}}\left(1-\frac{\log\det\left(I+\frac{g_{mimo}K_{x_{1}}g_{mimo}^{H}}{\sigma_{e}^{2}}\right)}{H(W_{t}^{\prime \prime })}\right). \end{array}$$

From (8), (35) and (A29), Δ≥δ in (15) is guaranteed if we have the following:

(A30) $$\begin{array}{c} \sigma^{2}\geq \underset{\mathrm{Secrecy}\mathrm{level}\mathrm{of}\mathrm{PLS}}{\underset{︸}{\max_{t\in \{1,\ldots ,T\}}\left[\frac{D\cdot 2^{\frac{2}{q(1-\delta )}\log\det\left(I+\frac{g_{mimo}K_{x_{1}}g_{mimo}^{H}}{\sigma_{e}^{2}}\right)}-S_{\ell }\sigma_{w,t}^{2}}{K}\right]}}. \end{array}$$

With the assumption that the edge server has an imperfect CSI of the eavesdropper’s channel, combining (A30) and Definition 3, (A30) can be re-written by the following:

(A31) $$\begin{array}{c} \sigma^{2}\geq \underset{\mathrm{Secrecy}\mathrm{level}\mathrm{of}\mathrm{PLS}}{\underset{︸}{\max_{t\in \left\{1,\ldots ,T\right\},\Delta g_{mimo}}\left[\frac{D\cdot 2^{\frac{2}{q(1-\delta )}\log\;\det\left(I+\frac{{\hat{g}}_{mimo}K_{x_{1}}{\hat{g}}_{mimo}^{H}}{\sigma_{e}^{2}}\right)}-S_{\ell }\sigma_{w,t}^{2}}{K}\right]}}, \end{array}$$

where ${\hat{g}}_{mimo}=g_{mimo}-\Delta g_{mimo}$. Then, combining (A3) and (A31), (16) in Theorem 1 is obtained.

The proof of Theorem 1 is completed.
